# Engineered Brain‐Targeted Exosomes Delivering FGF1 for Sustained Glycemic Regulation and Multitarget Neurovascular Protection in Diabetic Stroke

**DOI:** 10.1002/advs.77064

**Published:** 2026-08-11

**Authors:** Bixin Shen, Chengxiang Zhang, Junhui Wang, Xichong Zhong, Shihao Chen, Xue Wang, Xiaokun Li, Li Lin

**Affiliations:** ^1^ State Key Laboratory of Macromolecular Drugs and Large‐scale Preparation School of Pharmaceutical Sciences Wenzhou Medical University Wenzhou Zhejiang China; ^2^ Oujiang Laboratory (Zhejiang Lab for Regenerative Medicine, Vision and Brain Health) School of Pharmaceutical Sciences Wenzhou Medical University Wenzhou Zhejiang China

**Keywords:** brain targeted drug delivery system, fibroblast growth factor, ischemic stroke, neural targeting, type 2 diabetes

## Abstract

Diabetic stroke is characterized by a hyperglycemic and pro‐inflammatory microenvironment that exacerbates neurovascular dysfunction. However, the blood–brain barrier (BBB) remains a formidable obstacle, restricting the delivery of most therapeutic molecules. To address this, we developed a non‐invasive treatment strategy using engineered exosomes. Specifically, we fabricated FGF1‐loaded exosomes functionalized with the rabies virus glycoprotein (RVG) peptide (FGF1‐RVG Exo). This platform facilitates selective, neuron‐targeted delivery of FGF1 to the ischemic penumbra via RVG‐mediated transcytosis. In a diabetic stroke mouse model, FGF1‐RVG Exo exhibited superior pharmacological efficacy compared to free FGF1, achieving robust therapeutic outcomes with only once‐weekly administration. Notably, a single dose during the acute phase elicited a sustained hypoglycemic effect lasting up to two weeks and effectively ameliorated systemic insulin resistance. Locally, the accumulation of exosomes within the lesion led to a significant reduction in infarct volume and cell apoptosis, while promoting neovascularization and the recovery of motor and cognitive functions. This brain‐targeted strategy achieves a peripheral–central synergistic modulation, addressing the multi‐target requirements of diabetic stroke management. Collectively, our findings provide a novel paradigm for treating diabetic ischemic stroke and a potent strategy for the targeted delivery of growth factors to the central nervous system.

## 1 Introduction

According to the Global Burden of Disease 2021 estimates, stroke has emerged as the second leading cause of mortality worldwide, accounting for approximately 7 million deaths annually and imposing a staggering socioeconomic burden globally [1]. Notably, studies have emphasized that current stroke prevention strategies remain insufficiently effective to halt‐let alone reverse, the rapidly growing burden of stroke [2]. In China, diabetic stroke represents a major cause of death and disability among adults. type 2 diabetes (T2D) is a well‐established independent risk factor for ischemic stroke; notably, approximately 33% of stroke patients suffer from comorbid diabetes, which markedly elevates the risk of recurrence and worsens clinical outcomes [3]. The therapeutic management of diabetic stroke necessitates a multifaceted approach that transcends simple glycemic control to include the mitigation of acute cerebrovascular injury, promotion of angiogenesis, and restoration of neurological functions. However, developing pharmaceutical interventions that simultaneously address these complex pathological features remains a formidable challenge. To date, tissue plasminogen activator ‐mediated thrombolysis remains the only FDA‐approved pharmacological treatment for acute ischemic stroke [4, 5]. Several cardiovascular outcome trials have demonstrated that glucagon‐like peptide‐1receptor agonists (e.g., liraglutide, dulaglutide, semaglutide) reduce major cardiovascular events, with some evidence of beneficial effects on stroke [6, 7]. In contrast, sodium‐glucose cotransporter 2 inhibitors have shown generally neutral or inconsistent benefits in stroke treatment [8, 9]. Consequently, there is a critical unmet clinical need for multi‐target therapeutic agents capable of integrating neuroprotection with metabolic regulation.

Neurotrophic factors, particularly fibroblast growth factors (FGFs), have recently gained prominence for their potent roles in post‐stroke neural recovery [10, 11, 12, 13]. Among the 22 identified members of the FGF family, FGF1—a 17–18 kDa polypeptide‐stands out due to its pleiotropic pharmacological profile [14]. Beyond its canonical roles in wound healing and angiogenesis, FGF1 acts as a potent insulin sensitizer that regulates glucose homeostasis and enhances insulin sensitivity without the risk of hypoglycemia [15, 16]. In addition, FGF1 exhibits multiple functions, including neuroprotection, anti‐inflammatory activity, neurogenesis [17, 18, 19, 20], positioning it as a promising candidate for treating diabetic stroke. Our previous work demonstrated that peripheral administration of recombinant FGF1 could reduce infarct volume and blood glucose in diabetic mice [21], while intranasal delivery modulated microglia/macrophage polarization via the Nrf2 and NF‐κB pathways to attenuate brain injury [19]. Nevertheless, the clinical translation of FGF1 is severely hindered by its macromolecular nature, poor stability, and inability to cross the blood–brain barrier（BBB） [22]. Given that FGF1 belongs to the paracrine subfamily of FGFs, it is necessary to develop an appropriate brain‐targeting delivery system to deliver effective concentrations of FGF1 to the brain injury site in order to exert its local neuroprotective effects. Thus, developing a brain‐targeted delivery system is imperative to achieve therapeutic concentrations at the injury site while minimizing systemic side effects.

The BBB represents a formidable obstacle, impeding the majority of therapeutic agents from accessing the brain parenchyma. Current biomaterial‐based interventions aim to reconstruct the stroke microenvironment by delivering regenerative factors into the lesion, thereby activating endogenous neurogenesis and angiogenesis [23]. Exosomes are nanometer‑sized, naturally derived vesicles that carry a range of bioactive compounds (including nucleic acids, lipids, and proteins) from their parent cells [24, 25, 26, 27]. Even without functionalization, exosomes exhibit intrinsic properties that facilitate BBB penetration, highlighting their considerable potential as brain‑targeted drug carriers [28, 29]. To augment their specificity, surface modification with Rabies Virus Glycoprotein (RVG) peptides has been widely employed, as RVG specifically binds to nicotinic acetylcholine receptors (nAChRs) on the surface of neurons. However, current applications of exosome‑derived nanocarriers have predominantly focused on the loading of small‑molecule drugs and endogenous molecules into vesicles [30]. The challenge of orchestrating the targeted delivery of exogenous therapeutic proteins—such as growth factors—remains an under‐addressed yet critical frontier in neuro‐regenerative medicine.

Inspired by the inherent biocompatibility of exosomes and the neuronal affinity of RVG peptides, we hypothesized that an engineered exosomal platform could enable the targeted delivery of FGF1 to the injured brain. In this study, we developed a brain‐targeted growth factor delivery system in which FGF1 was loaded onto the surface of engineered RVG‑modified exosomal membranes (termed “FGF1‐RVG Exo”) (Figure 1). Our results confirmed the successful loading of FGF1, which significantly enhanced its BBB penetration efficiency. The brain‑targeting property of RVG exosomes facilitated the deep infiltration of FGF1 into both cerebral infarct areas and glucose homeostasis centers, thereby augmenting its neuroprotective and antihyperglycemic efficacy. This non‐invasive approach effectively elevated FGF1 concentrations within the ischemic lesion, ensuring sustained bioactivity. Consequently, this system markedly improved the therapeutic outcomes of FGF1 in a diabetic stroke mouse model, achieving superior efficacy at lower dosages and reduced administration frequencies.

**FIGURE 1:**
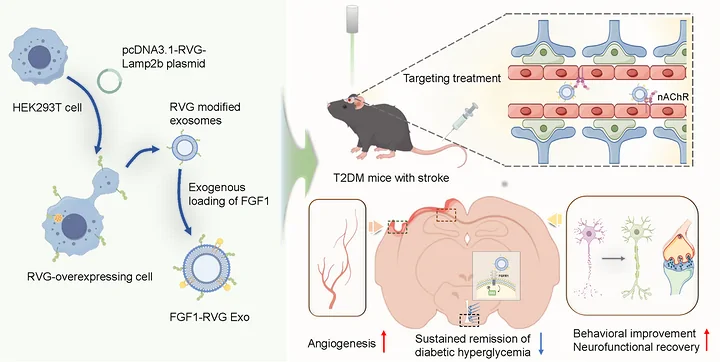
Schematic illustration of the treatment strategy using FGF1‑RVG Exo in a mouse model of diabetic stroke. Left: Preparation of FGF1‑loaded RVG‑modified exosomes. Right: Following intravenous administration, FGF1‐RVG Exo undergo RVG‐mediated transcytosis across the BBB to specifically target neurons. This targeted delivery elicits multifaceted therapeutic effects, including sustained glucose homeostasis, neuroprotection, and promoted angiogenesis.

## 2 Results

### 2.1 Diminished FGF1 Expression in T2D Patients and T2D Mice

To investigate the role of FGF1 in the pathogenesis of diabetic stroke, we first analyzed *FGF1* expression profiles of human normal tissues using RNA‐seq data from the HPA database (Figure S1) and found that *FGF1* was highly expressed in the cerebral cortex. We further examined FGF family expression levels in the GSE26168 dataset, which contains blood samples from patients with impaired fasting glucose (IFG) and T2D. The hierarchical cluster heatmap indicated that among the 22 FGF genes, only *FGF1* was associated with diabetes and IFG (Figure 2A). Compared to healthy controls, serum *FGF1* levels were decreased in IFG and T2D patients. Moreover, KEGG analysis of downregulated differentially expressed genes (DEGs) in T2D revealed enrichment in FGF‑related signaling pathways, including the cAMP, neurotrophin, and PI3K‑AKT pathways (Figure 2B). To further validate the protein‐level changes of FGF1 in disease, we established a T2DM mouse model by feeding a high‐fat diet combined with intraperitoneal injection of streptozotocin (STZ). We then compared FGF1 levels in the cerebral cortex and hippocampus of three groups of mice: normoglycemic mice, high‑fat‑fed mice, and T2DM mice. The results demonstrated a decrease in brain FGF1 levels in subjects with impaired glucose tolerance and T2DM, consistent with the bioinformatics findings (Figure 2C–E).

**FIGURE 2:**
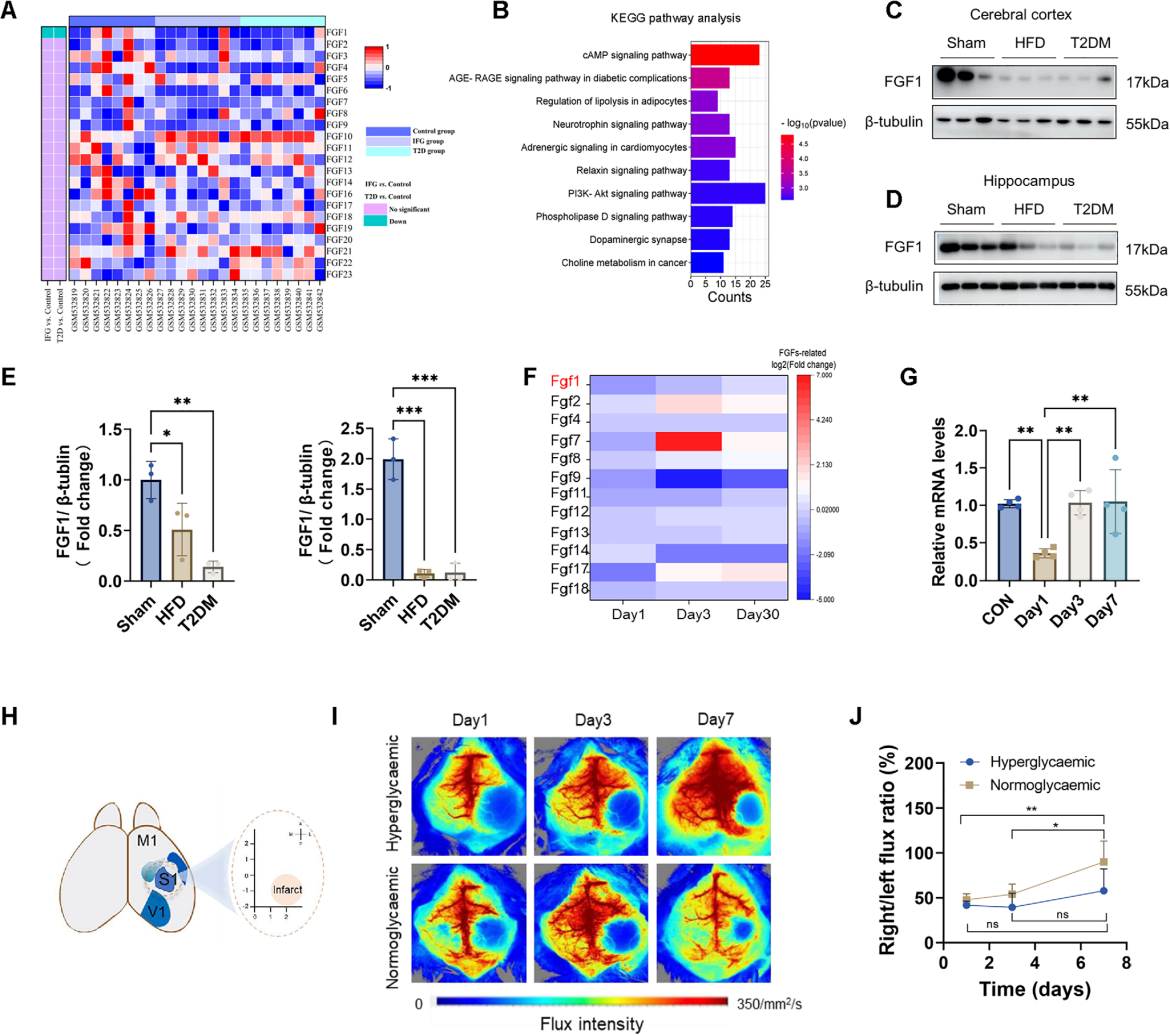
The expression level of FGF1 decreases in type 2 diabetes mellitus. (A) Heat maps obtained by analyzing 856 differential genes obtained from GSE26168 database. (B) KEGG enrichment analysis results of downregulated DEGs in GSE26168 database. (C,D) Expression of FGF1 in mouse cerebral cortex and hippocampal tissue. (E) Statistics for Figure 2C,D. Data are presented as mean ± SD; Statistical significance was assessed using one‑way ANOVA. ^*^p <0.05, ^**^p <0.01, ^***^ *p* < 0.001. (F) Analysis of RNA transcriptomic sequencing data from brain endothelial cells of post‐stroke mice. (G) The content of *Fgf1* mRNA in brain tissue after PT injury; Data are presented as mean ± SD; Statistical significance was assessed using one‐way ANOVA. ^**^p <0.01. (H) Schematic diagram of the position of the mold in the stroke. (I) Representative doppler cerebral blood flow diagrams were shown at day1,3 and 7 post‐stroke. (J) Blood flow data are presented as mean ± SD (n = 3). Statistical significance was assessed using two‑way analysis of variance (ANOVA). ^*^p <0.05, ^**^p <0.01, ns, not significant.

To further evaluate FGF expression in ischemic stroke, we analyzed RNA sequencing data from a brain endothelial cell transcriptome resource under healthy and ischemic stroke conditions. Specifically, we assessed gene expression changes in mouse peri‑infarct endothelial cells at 1, 3, and 30 days after middle cerebral artery occlusion compared with sham‑operated controls. We found that *Fgf1* expression was significantly downregulated at day1, and gradually increased at days 3 and 30 (Figure 2F). Furthermore, we measured cortical *Fgf1* levels in mice on days 1, 3, and 7 after stroke. The results confirmed a marked downregulation of *Fgf1* during the acute phase (Figure 2G), which was consistent with the sequencing results shown in Figure 2F. Collectively, these data provide molecular evidence that FGF1 expression is closely associated with the occurrence of ischemic stroke in patients with T2DM.

To investigate whether diabetic stroke leads to more severe neurovascular damage than normoglycemic stroke, we established a photothrombotic (PT) stroke model in mice with or without T2DM. Cortical blood flow was assessed using MESI, and neuronal functional recovery in the primary sensory cortex (Figure 2H) was evaluated after stroke. We first employed laser doppler flowmetry to measure blood flow perfusion recovery in the infarcted region at days 1, 3, and 7 post‐stroke (Figure 2I,J). Statistical analysis of perfusion on the ipsilateral and contralateral sides indicated that although infarct size in diabetic stroke mice did not differ significantly from that in normoglycemic mice by day 7, cerebrovascular perfusion recovered more slowly under hyperglycemic conditions. Specifically, in diabetic stroke mice, blood supply in the lesion area at day 7 showed no statistically significant difference compared with day 1, whereas normoglycemic mice exhibited a substantial increase in perfusion within the lesion area at day 7 relative to day 1.

Since the stroke lesion in this study targeted the cortical region governing sensory function in the forelimbs and hindlimbs, we further evaluated motor function deficits using multiple behavioral tests. In the grid‑walking test on day 7 after PT model induction, the rate of foot faults was significantly lower in hyperglycemic stroke mice than in normoglycemic stroke mice. Diabetic stroke mice also exhibited lower grip strength and higher modified Neurological Severity Scale (mNSS) scores. These results demonstrate that diabetic stroke mice show more severe motor impairment compared with normoglycemic stroke‑induced mice (Figure S2A–C).

### 2.2 Preparation and Characterization of Engineered Exosomes

To generate RVG‑modified exosomes (RVG‑Exos), we fused RVG to lysosomal‑associated membrane protein 2b (Lamp2b) and transfected HEK293T cells with the pcDNA3.1‐RVG‐Lamp2b‐GFP plasmid using polyethyleneimine (PEI), with GFP as a fluorescent reporter. High transfection efficiency was confirmed by fluorescence microscopy and flow cytometry (Figure 3A–C). We then monitored the biogenesis of extracellular vesicles in live cells. Lysosomes were pre‑stained with a green fluorescent marker, and the cell membrane was labeled with DiD dye. Under physiological conditions, the cell membrane undergoes continuous remodeling, leading to the formation of both inward and outward budding structures within minutes. These structures generate multivesicular bodies (MVBs) via endocytosis, which subsequently fuse with the plasma membrane to release exosomes (Figure 3D).

**FIGURE 3:**
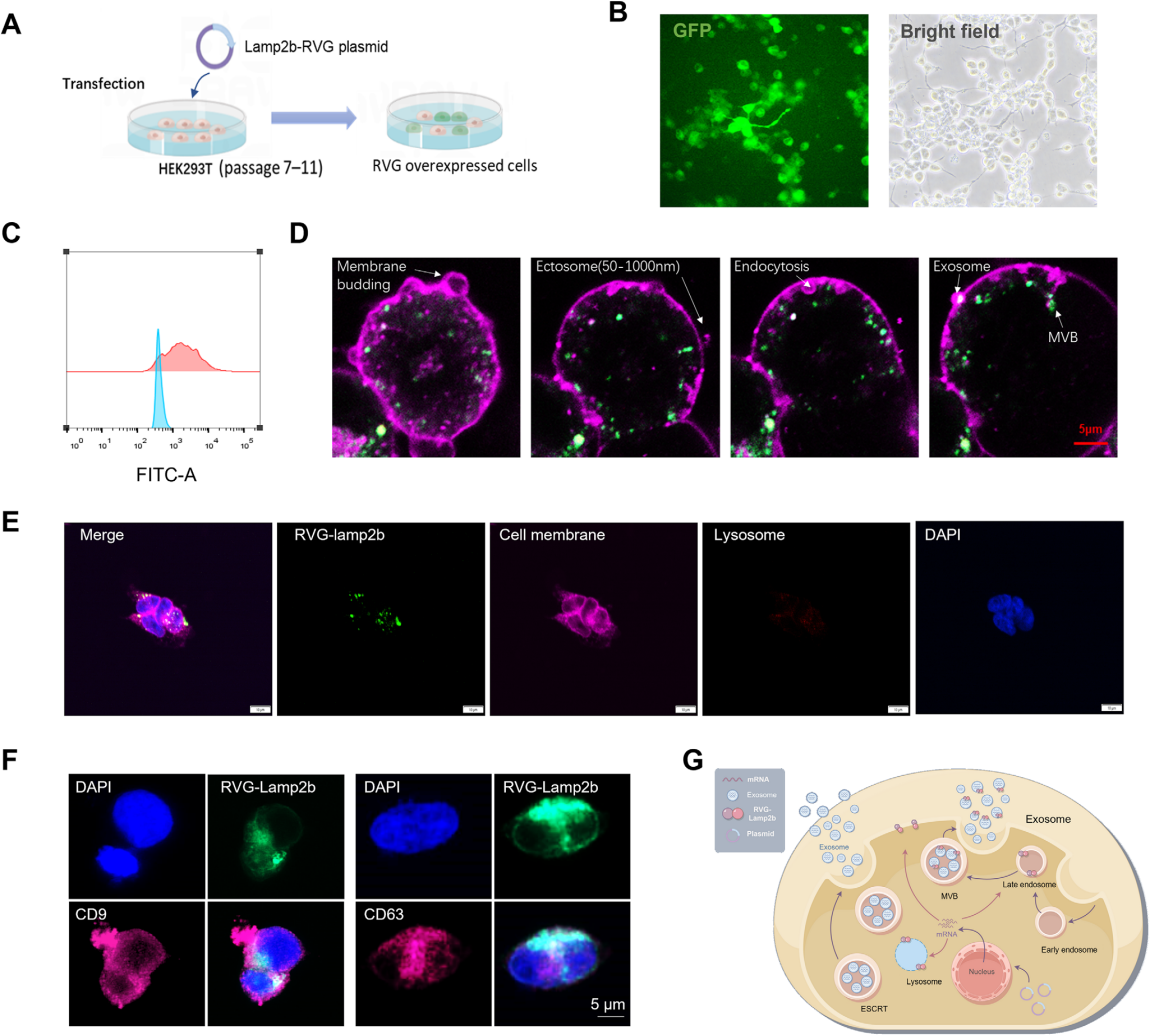
The formation of RVG‐modified exosomes. (A) Schematic showing RVG‐Lamp2b plasmids were transfected into the exosome‐donor cells (HEK293T) using PEI. (B‐C) Transfection efficiency was verified by fluorescence microscopy and flow cytometry. (D) Dynamic tracking of exosomes in HEK293T cells. (E) The colocalization of RVG‐Lamp2b with the plasma membrane and lysosomes. (F) The colocalization of RVG with exosome markers. (G) Schematic illustration of exosome formation.

We next examined the intracellular distribution of the transfected RVG protein. As shown in Figure 3E, the RVG protein primarily co‐localized with lysosomes and endosome‐like organelles, with minimal presence on the cell membrane. We then performed co‐localization staining for the RVG protein with the exosome markers CD9 and CD63, which revealed distinct distribution patterns: CD63 was specific to endosomes and lysosomes, whereas CD9 was mainly found on the cell membrane. The results demonstrated that the RVG protein showed more extensive co‐localization with intracellular endosomes and lysosomes (Figure 3F). In summary, the biogenesis process of exosomes comprised four principal stages: cargo sorting into the maturing MVBs, MVB transport, MVB fusion with the cell membrane, and release. The formation of MVBs, characterized by membrane budding and generation of intraluminal vesicles (ILVs), is considered a central step in exosome biogenesis. The transfection strategy also affected the cargo loading efficiency into exosomes. In this study, by fusing the RVG with the exosomal membrane protein Lamp2b, we achieved efficient endogenous loading of the RVG protein into exosomes. Based on the cargo sorting mechanism of exosomes (including ESCRT‐dependent pathways and ESCRT‐independent pathways), we found that fusing the target protein with plasma or lysosomal membranes provides a more effective approach for directing proteins into exosomes (Figure 3G).

To prepare RVG Exo, the cell culture medium was collected 48 h after transfection. The supernatant was then sequentially centrifuged at 3000×g for 30 min to remove apoptotic bodies, at 10 000×g for 60 min to remove large extracellular vesicles (EVs), and finally at 100 000×g for 120 min to pellet the exosomes. The obtained RVG‑Exo pellets were resuspended in sterile PBS buffer and stored at −80°C for further use (Figure 4A). Transmission electron microscopy (TEM) imaging confirmed that the exosomes displayed the characteristic tea‐saucer‐like morphology of vesicles (Figure 4B). RVG‑Exo showed a homogeneous particle size of approximately 120 nm (Figure 4C). Western blot analysis demonstrated the presence of the exosome signature proteins ALIX, CD63, CD9, Lamp2b and TSG101 in both native exosomes (Native‑Exo) and RVG‑Exo, whereas the endoplasmic reticulum protein calnexin was not detected (Figure 4D). Triton X‐100 is a nonionic surfactant that permeabilizes cell membranes at low concentrations and lyses them at higher concentrations. Adding a specific proportion of Triton X‐100 reagent to RVG Exo revealed changes in both the size distribution and particle concentration of exosomes, indicating high purity of the isolated exosomes (Figure S3). The Coomassie Brilliant Blue staining revealed that RVG Exo displayed protein expression profiles similar to those of Native‑Exo but distinct from total cellular protein (Figure 4E). We next examined the UV absorption properties of the exosomes (Figure 4F and Figure S4). The results showed that the RVG Exo had a higher nucleic acid content than Native‑Exo. Furthermore, the lipidomic analysis revealed no significant differences in total lipid intensity between RVG Exo and Native Exo (Figure 4G). Furthermore, the results of the lipidomic analysis demonstrated that the lipids present in RVG Exo mainly consisted of triglycerides (TG, 37%), phosphatidylcholine (PC, 17%), diglycerides (DG, 13%), ceramides (Cer, 9%), and phosphatidylethanolamine (PE, 5.6%) (Figure 4H,I). Notably, the total intensity of TG was higher in RVG‑Exo, whereas that of PC was lower than in Native‑Exo. These natural lipids help maintain the structure of RVG‑Exo, protect the encapsulated bioactive payload, and provide excellent biomembrane permeability, facilitating transmembrane delivery of the loaded drugs.

**FIGURE 4:**
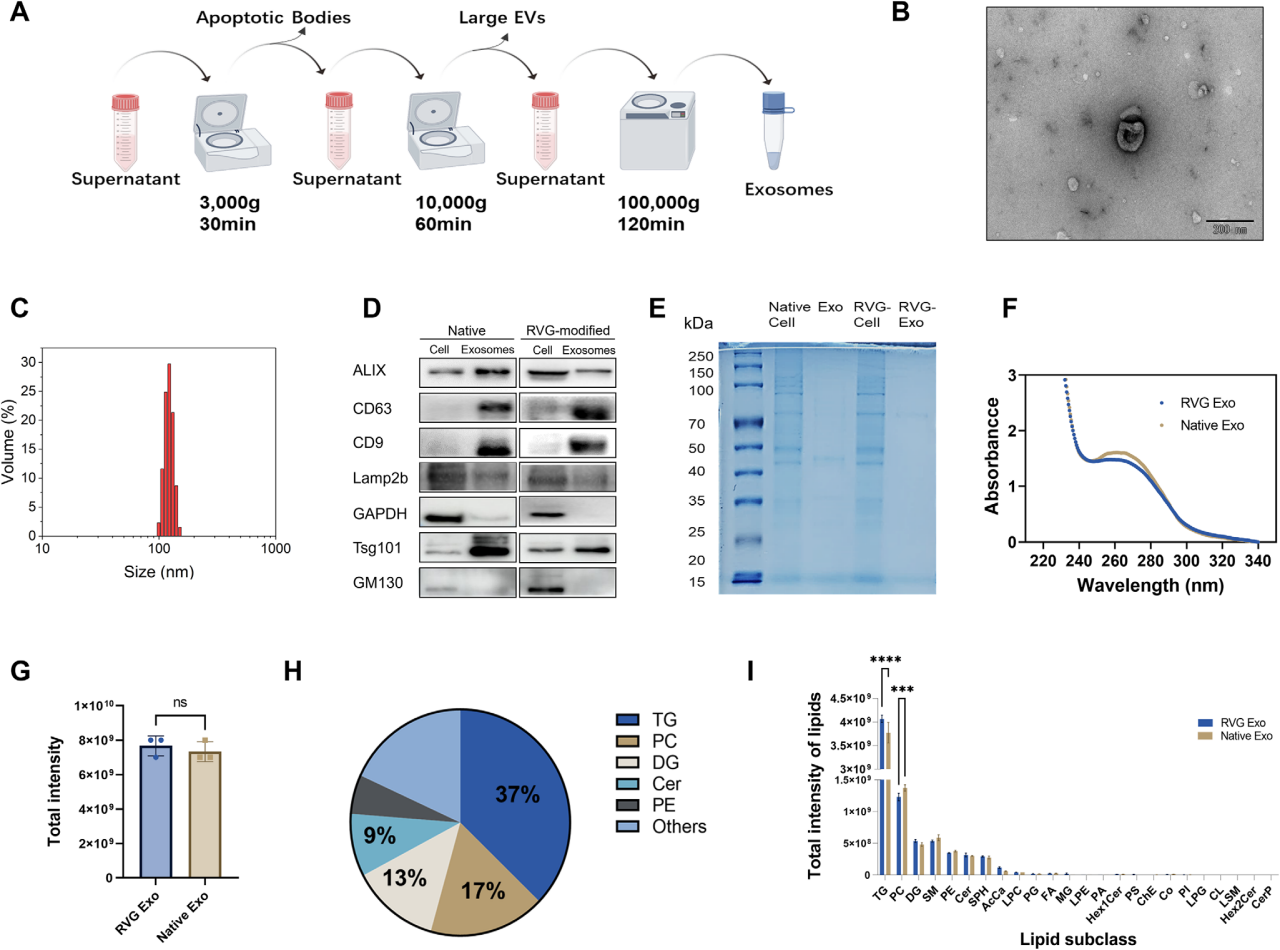
Preparation and characterization of exosomes. (A) Schematic diagram of the preparation process of exosomes. (B) TEM image of RVG Exo. Scale bar = 200 nm. (C) The size distribution of in RVG Exo. (D) Western blotting analysis of cells and exosomes. (E) The Coomassie Brilliant Blue staining of exosomes. (F) UV absorption spectrum of exosomes. (G) Total lipid intensity of exosomes. Statistical significance was assessed using a two‐tailed unpaired Student's t‐test. Data are presented as mean ± SD (n = 3). ns, not significant. (H,I) Category and quantities of the lipids in RVG Exo. TG: triglycerides, PC: phosphatidylcholine, DG: diglycerides, Cer: ceramides, PE: Phosphatidylethanolamine.

Before loading FGF1, it is essential to thoroughly evaluate the ability of RVG Exo to cross the BBB. To this end, we labelled the RVG Exo with DiD (a red fluorescent lipid‐labeled dye). Mice were administered 0.1 mL of PBS containing either 10^9^ DiD‑labeled exosomes or free DiD dye alone, and in vivo imaging was performed three hours after administration. Significant fluorescent intensity was detected in the brain of mice injected with RVG Exo (Figure S5). In contrast, mice injected with the same dose of free dye exhibited predominant fluorescence accumulation in peripheral tissues, with minimal fluorescence observed in the brain. Quantitative analysis further confirmed that RVG‑Exo penetrated deeply into the brain. These results provide strong evidence that RVG‑Exo possess the ability to cross the BBB.

### 2.3 Preparation and Characterization of FGF1‐RVG Exo

Given that in vivo imaging experiments demonstrated that RVG‑modified exosomes effectively cross the intact BBB, we propose that engineered RVG exosomes hold great potential as carriers for brain drug delivery. In preparing FGF1‑loaded exosomes, we initially considered engineering HEK293T cells to overexpress FGF1 and collecting the supernatant to obtain FGF1‑loaded exosomes, based on previous studies [31, 32]. However, after co‐transfecting both the pcDNA3.1‐FGF1‐RFP plasmid and the pcDNA3.1‐RVG‐Lamp2b‐GFP plasmid, we observed that the proportion of double‑positive cells decreased to approximately 50%, indicating low transfection efficiency (Figure S6). Furthermore, immunofluorescence co‑localization analysis revealed that FGF1 was predominantly localized to the cytoplasm at high expression levels in transfected cells (Figure S7A). Subsequent Western blot detection of FGF1 content in the isolated exosomes indicated low enrichment of FGF1 in FGF1‑Exo (Figure S7B). ELISA results further confirmed that the FGF1 content of the exosomes was only 12.1 ± 2.05 µg/mL (Figure S7C). To further investigate the reason for the low sorting efficiency of FGF1 into exosomes derived from highly expressing donor cells, we performed immunofluorescence co‑localization staining of FGF1 with the exosome marker proteins CD9 and CD63. The results showed that FGF1 did not exhibit high co‑localization with CD63 within cells (Figure S7D). This suggests that not all cytoplasmic proteins are efficiently sorted into exosomes during the biogenesis process.

Given the low efficiency of endogenous FGF1 loading, we next explored the use of exosomes as carriers to achieve efficient loading and brain‑targeted delivery of FGF1. An exploratory approach was adopted to compare three methods for preparing FGF1‑loaded RVG exosomes (FGF1‑RVG Exo): incubation, freeze‑thaw cycling, and ultrasonication (Figure 5A). After each treatment, a gentle washing procedure was employed to remove unencapsulated FGF1, ensuring the integrity and efficacy of the encapsulated product. Briefly, 60 µg of protein‐equivalent exosomes were mixed with 15 µL of 2 mg mL^−1^ FGF1 in PBS buffer. For the incubation method, the mixture was conducted at 37°C for 12 h. During this process, a portion of FGF1 was initially adhered to the surface due to hydrophobic interactions, while part of them was encapsulated within exosomes. For freeze‐thaw method, the mixture underwent three consecutive from −80°C to 37°C cycles. For the ultrasonication method, 0.5 mL of PBS was added to the mixture, followed by five cycles of sonication (30 s per cycle at 20% amplitude). After each treatment, the mixture was then transferred into a centrifugal filter with a molecular weight cutoff (MWCO) of 10 kDa, followed by centrifugation at 3500 rpm for 20 min to remove any unencapsulated FGF1. Consequently, the FGF1‐RVG Exo was proficiently retained in the upper chamber of the centrifugal concentrator, which represented our target product, as depicted in Figure 5A. Subsequently, Western blot analysis was performed to measure FGF1 levels in the upper and lower filtrate layers of the product after the third filtration process. Results indicated that both the incubation method and freeze‐thaw method could form relatively stable exosome‐encapsulation systems, with significantly higher FGF1 concentrations in the upper chamber than in the lower chamber. In contrast, the ultrasonication method exhibited relatively lower encapsulation efficiency (Figure 5B). The result of the native page analysis showed that the formation of the FGF1‐RVG Exo did not affect the charge or structural properties of FGF1 (Figure 5C). We further measured the encapsulation rates of FGF1 in FGF1‐RVG Exo prepared by three methods. Quantitative encapsulation rate analysis revealed that, among the three methods, incubation achieved the highest encapsulation rate, with RVG‑Exo and native exosomes (Native‑Exo) exhibiting rates of 54.3% ± 4.9% and 43.6% ± 3.1%, respectively. The ultrasonication method yielded the lowest encapsulation rate, with RVG‑Exo at 26.1% ± 4.9% and Native‑Exo at only 11% ± 4.2% (Figure S8).

**FIGURE 5:**
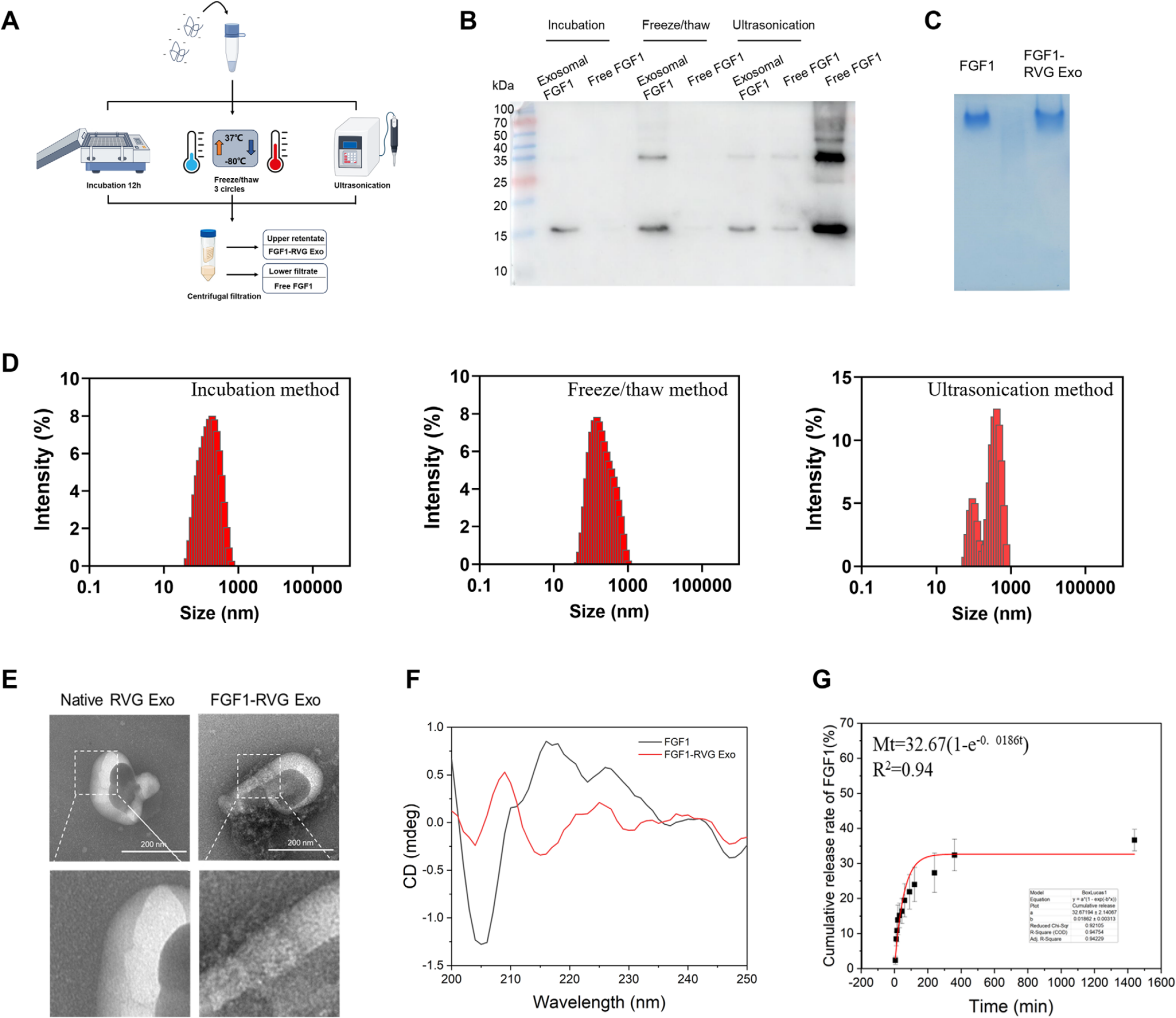
Preparation and Characterization of FGF1‐RVG Exo. (A) Schema illustrates the preparation of FGF1‐RVG Exo using three different methods. (B) Western blot analysis of FGF1 in upper and lower chambers of filtration. (C) Native page of FGF1‐RVG Exo. (D) DLS measurements for hydrodynamic diameter of FGF1‐RVG Exo prepared by different methods. (E) Representative transmission electron microscopy images of FGF1‐RVG Exo. (F) Circular dichroism spectroscopy of FGF1‐RVG Exo. (G) In vitro release kinetics of FGF1 from FGF1‐RVG Exo at 37°C.

The physicochemical properties of intact native exosomes and FGF1‑RVG Exo were characterized by dynamic light scattering (DLS). The hydrodynamic diameter and polydispersity index (PDI) of RVG Exo were 174.1 ± 10.5 nm and 0.45, respectively (Figure 5D, Figure S9A,B). After FGF1 loading, the particle size of FGF1‑RVG Exo increased depending on the preparation method: 213.4 ± 61.6 nm for the freeze‐thaw method, 189.6 ± 9.6 nm for the incubation method, and 62.9 ± 9.9 nm for the ultrasonication method. The PDI values of the loaded exosomes were similar (approximately 0.5), indicating that FGF1 loading slightly increased the hydrodynamic size, except for the ultrasonication method, where the markedly smaller size suggested possible damage to the exosomal membrane structure. The zeta potential of RVG Exo was measured at −11.3 ± 0.93 mV, while that of the FGF1‐RVG Exo showed an increase (−10.8 ± 2.3 mV in the freeze‐thaw method, −8.8 ± 1.5 mV in the incubation method, and −7.9 ± 1.5 mV in the ultrasonication method) (Figure S9C). The increase in zeta potential can be attributed to the neutral nature of FGF1, which reduces the surface charge density of the exosomes. Based on a comprehensive analysis of DLS results, we concluded that among the three methods, the freeze–thaw method resulted in higher polydispersity and lower drug loading capacity, whereas the ultrasonication method tended to disrupt the exosomal membrane structure, making it difficult to obtain homogeneous drug‑loaded formulations. Therefore, the incubation method was selected for subsequent experiments. To optimize loading efficiency, we investigated the effect of varying the mixing ratio of exosomes to FGF1. Exosomes were incubated with FGF1 at protein‐to‐membrane protein ratios of 1:1, 1:2, and 1:4. The encapsulation efficiency was assessed by Western blot, and the results demonstrated that a ratio of 1:2 (FGF1: exosome) yielded the highest FGF1 loading (Figure S10A).

TEM was then used to determine whether the morphology of exosomes was maintained after FGF1 encapsulation. As shown in Figure 5E, FGF1‑RVG Exo retained a spherical shape similar to that of native exosomes, indicating that its structural integrity had been preserved. In contrast to blank RVG‐Exo, the outer membrane of the RVG‐Exo no longer exhibited a completely smooth lipid bilayer structure after FGF1 loading; instead, FGF1 proteins were embedded within the membrane. Some of the proteins inserted partially into the lipid bilayer, while the majority bound to the surface of the membrane proteins, forming a complex‐like structure. The successful preparation of FGF1‑RVG Exo was further confirmed by confocal imaging, which showed co‑localized fluorescence of exosomes and FGF1 (Figure S10B). Circular dichroism spectroscopy revealed that the secondary structure of FGF1 remained unchanged upon exosome loading (Figure 5F). In vitro drug release kinetic analysis was conducted at 37°C for the FGF1‑RVG formulation. The results indicated that FGF1 loaded onto exosomes exhibited a sustained‑release profile, with the release kinetics fitting a first‑order model (Figure 5G and Figure S10C).

### 2.4 Enhanced Penetration of FGF1‐RVG Exo Across the Intact BBB Depends on RVG Modification

To evaluate the brain‑targeting ability of FGF1‑RVG Exo after stroke, we induced PT stroke in nude mice and injected various fluorescence‑labeled exosomes via the tail vein. Fluorescence signal distribution was monitored at 3 h post‑injection using an IVIS Spectrum system (Figure 6A and Figure S11). In vivo imaging revealed that both native exosomes (Native Exo) and RVG Exo effectively accumulated in the brain, whereas no discernible fluorescence signal was observed in the PBS‑treated group. To further evaluate the organ distribution of RVG Exo, we performed ex vivo imaging of major organs. As shown in Figure S11, RVG modification significantly increased brain accumulation (consistent with Figure 6A). Meanwhile, both Native Exo and RVG Exo showed relatively higher accumulation in the liver, lung, and spleen compared with other peripheral organs, likely due to uptake by the mononuclear phagocyte system and the intrinsic particle size of exosomes. However, no statistically significant differences in liver, lung, or spleen accumulation were observed between Native Exo and RVG Exo. Notably, only weak fluorescence signals were detected in the heart and kidney, indicating that RVG Exo do not preferentially accumulate in these organs. Brain sections were further examined by confocal laser scanning microscopy (CLSM) to assess fluorescence distribution. Owing to their targeting ability, the signals of RVG Exo were detected in both the boundary and the core of the damaged area. In contrast, mice injected with the same dose of free dye exhibited fluorescence predominantly around the lesion boundary, with no detectable signal in the core region (Figure 6B and Figure S12). To investigate whether exosomes could deliver the loaded FGF1 to the brain, FGF1 was labeled with a green fluorescent dye. After injection, mice were euthanized at 3 h, and brain tissues were collected for sectioning to observe fluorescence distribution. The results demonstrated that RVG Exo not only increased FGF1 levels within cerebral blood vessels but also enabled FGF1 to reach the brain parenchyma beyond the vascular compartment, particularly in regions surrounding the injured vasculature (Figure S13).

**FIGURE 6:**
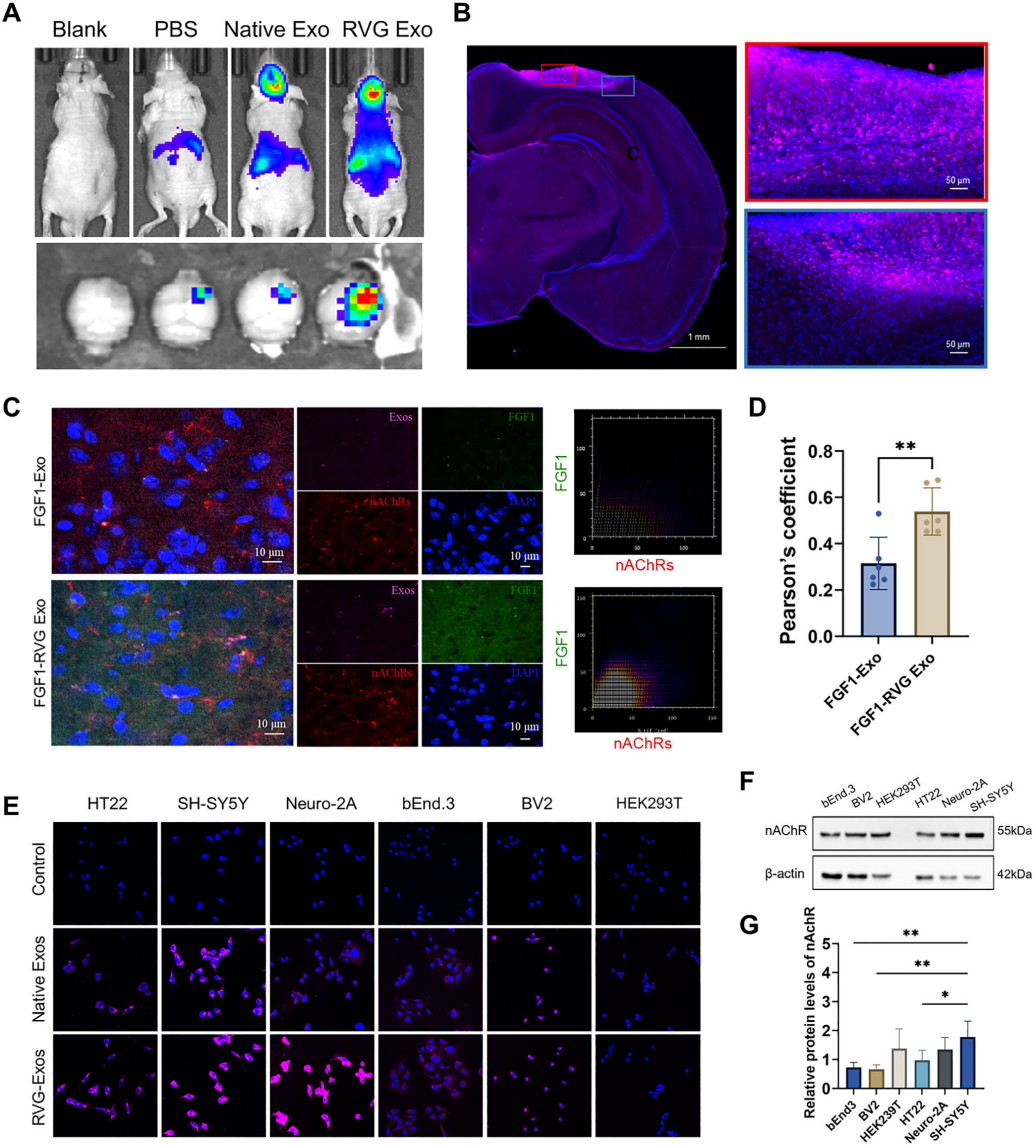
Determination and analysis of penetrative performance of FGF1‐RVG Exo through BBB. (A) The distribution of RVG‐EVs in mice after PT modeling. (B) Representative confocal fluorescent microscopy images of the brain section with i.v. injections of fluorescent‐labeled exosomes. (C,D) The colocalization of FGF1‐RVG Exo in nAChRs‐expressed cells. Statistical significance was assessed using a two‐tailed unpaired Student's t‐test. Data are presented as mean ± SD (n = 3). ^**^p <0.01. (E) Uptake behavior of FGF1‐RVG Exo in different types of cells. (F‐G) Western blot analysis of nAChRs expression in different types of cells. n = 3, Data are shown as mean ± SD. Statistical significance was assessed by one‐way ANOVA followed by Tukey's multiple comparisons test. ^*^p <0.05; ^**^p <0.01.

We next investigated the mechanism by which FGF1‑RVG Exo crosses the BBB. The neuronal targeting efficacy of RVG‑modified exosomes (RVG‑Exo) was validated both in vitro and in vivo, based on the reported ability of RVG peptides to interact with nAChRs on endothelial cells [33]. To assess this, we compared the co‑localization of native exosomes (Native Exo) and RVG‑Exo with nAChRs in brain tissue. Fluorescence staining and Pearson's correlation coefficient analysis revealed that the RVG‑Exo group exhibited significant co‑localization with nAChRs, whereas the Native Exo group showed no such co‑localization. These results indicate that, although Native Exo achieved higher brain penetration efficiency than the PBS group through passive targeting and the intrinsic transmembrane ability of exosomes, its performance was inferior to that of RVG‑Exo. RVG‑Exo not only facilitates transport across the BBB but also enables active targeting of nAChRs, thereby achieving specific cellular delivery (Figure 6C,D).

We further compared the specific targeting of RVG‑Exo to different cell types, including neuronal cells, microglia, bEnd.3 endothelial cells, and HEK 293T cells. As shown in Figure 6E and Figure S14, the uptake of FGF1‑RVG‑Exo by SH‑SY5Y, HT22, and Neuro‑2A cells was increased by 2.2‑fold, 3.8‑fold, and 14‑fold, respectively, compared with that of Native‑Exo. This enhanced uptake can be attributed to the heterogeneous surface expression of nAChRs (Figure 6F,G). Collectively, these results demonstrate the neuronal‑targeting capability of FGF1‑RVG‑Exo.

### 2.5 Sustained Remission of Hyperglycemia by Central and Peripheral Actions of FGF1‐RVG Exo

Having established the neuronal‑targeting capability of FGF1‑RVG Exo, we next validated its therapeutic efficacy in treating diabetic stroke. We first evaluated its hypoglycemic effect in a diabetic mouse model. Diabetic mice were divided into four groups: PBS, FGF1, FGF1‐Exo, and FGF1‐RVG Exo. The glucose‐lowering effects of each treatment were assessed within 24 h following a single tail vein injection. All three FGF1‐loaded formulations demonstrated effective hypoglycemic effects. Notably, the FGF1‐RVG Exo group exhibited significant hypoglycemic effects as early as 3 h post‐administration, whereas the other two groups reached comparable levels of hypoglycemic effects by 12 h post‐administration (Figure 7A).

**FIGURE 7:**
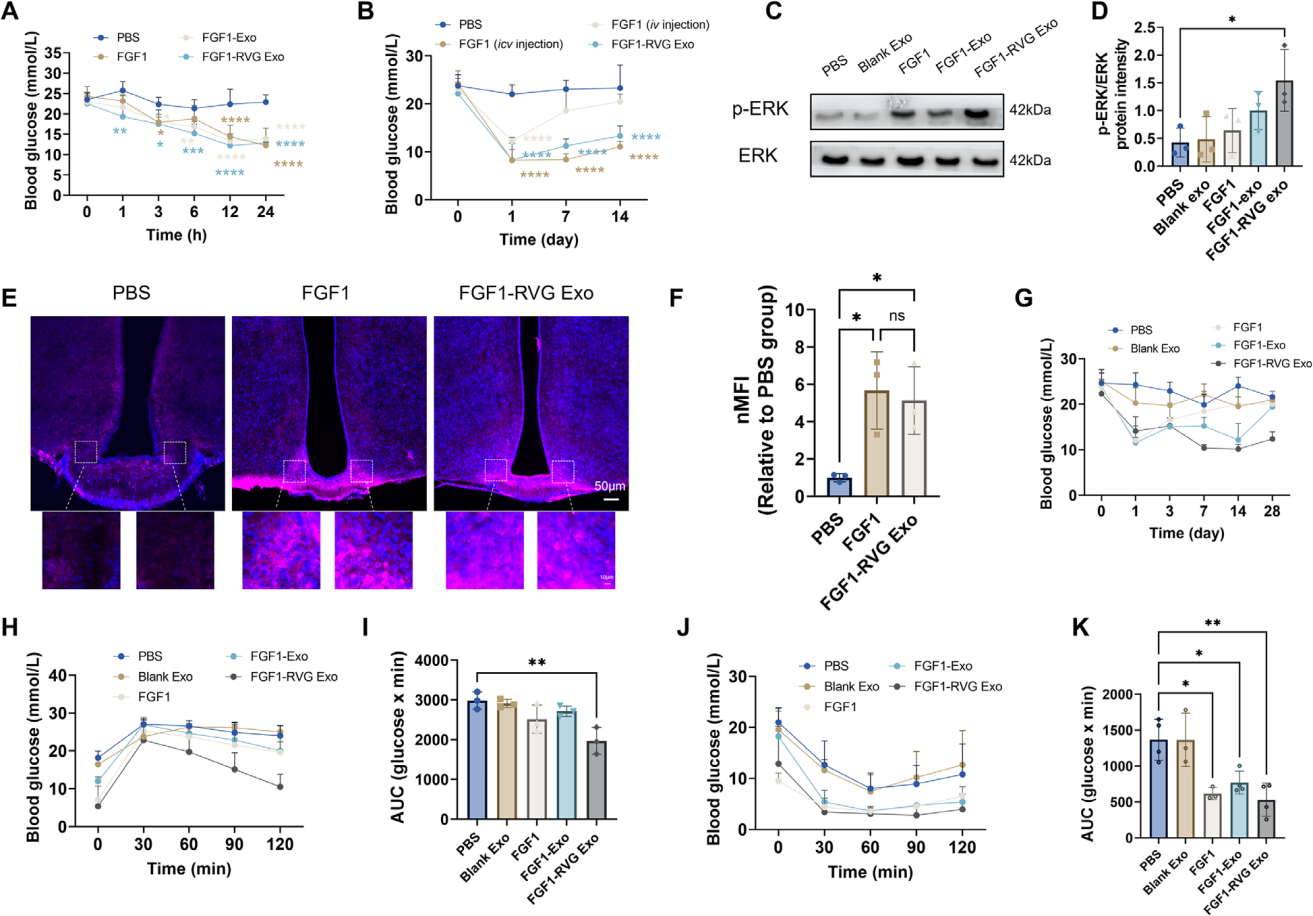
Sustained remission of hyperglycemia induced by both central action and peripheral action of FGF1‐RVG Exo. (A) Blood glucose levels in diabetic stroke mice within 24 h after administration of PBS, FGF1, FGF1‐Exo and FGF1‐RVG Exo (0.5 mg kg^−1^, i.v.). Data are presented as mean ± SD (n = 3–4 per group, ^*^ *p* <0.05, ^**^ *p* <0.01, ^***^ *p* <0.001, ^****^ *p* <0.0001). (B) Blood glucose levels in diabetic stroke mice after a single dose of PBS, FGF1 (0.5 mg kg^−1^, i.v.), FGF1 (3 µg, i.c.v.), and FGF1‐RVG Exo (0.5 mg kg^−1^, i.v.). Data are presented as mean ± SD (n = 3–4 per group, ^****^ *p* <0.0001). (C) WB analysis of p‐ERK and ERK expression in hypothalamic tissues excised from mice after different treatments. (D) Quantification of protein levels by group based on WB results. n = 3, ^*^ *p* <0.05. (E,F) Immunofluorescence of hypothalamic pERK after drug administration at the indicated time in T2DM mice. The quantification of the MFI (normalized to the PBS group) is shown on the right. Scale bars = 50 µm. (G) Changes in blood glucose of mice in different groups after treatment. (H,I) Changes in blood glucose and AUC of mice subjected to ipGTT 28 days after treatment. (J,K) Changes in blood glucose and AUC of mice subjected to ipITT 28 days after treatment. (A), (B), (G) are analyzed by two‐way RM ANOVA; (D), (F), (I), (K) are analyzed by one‐way ANOVA; ^*^ *p* <0.05; ^**^ *p* <0.01; ^***^ *p* <0.001; ^****^ *p <*0.0001 vs. corresponding PBS group. ns, not significant.

We next assessed the ability of FGF1‑RVG Exo to achieve sustained glycemic control. Previous studies have confirmed that a single intracerebroventricular (i.c.v.) injection of FGF1 can achieve hypoglycemic effects lasting over 14 days. Given that RVG‐modified exosomes enhance the brain penetration efficiency of FGF1, we sought to investigate whether a single dose of FGF1‑RVG Exo could also induce long‑lasting hypoglycemia. Diabetic mice were divided into PBS, i.v. injection of FGF1, i.c.v. injection of FGF1, and i.v. injection of FGF1‐RVG Exo. The dosage for intravenous administration of free FGF1 and FGF1‑RVG Exo was 0.5 mg kg^−1^, while the dosage for i.c.v. injection was 3 µg. The results showed that both i.c.v. injection of FGF1 and intravenous FGF1‐RVG Exo achieved hypoglycemic effects lasting up to two weeks, whereas a single intravenous dose of free FGF1 maintained hypoglycemic effects for only a few days (Figure 7B).

The hypothalamus has been identified as the key brain region responsible for the central effect of FGF1 [34]. To further evaluate whether the sustained hypoglycemic response of FGF1‐RVG Exo correlates with the activation of central FGF1 effects, diabetic mice were divided into PBS, blank Exo, FGF1, FGF1‐Exo, and FGF1‐RVG Exo groups. Mice were euthanized 3 h after intravenous administration (based on the results of Figure 7A). Western blot analysis was performed to detect pERK levels in hypothalamic tissue. Simultaneously, brain tissue sections were prepared to visualize pERK expression in the third ventricle of the hypothalamus. The Western blot analysis results showed that pERK expression in the FGF1‐RVG Exo group was significantly elevated compared with the PBS group (Figure 7C,D). Immunohistochemical staining of pERK was performed on brain sections from the FGF1 and FGF1‐RVG Exo groups, with matched sections photographed under identical conditions. A robust increase in pERK immunostaining was detected in the arcuate nucleus (ARC) following both i.c.v. FGF1 injection and i.v. FGF1‐RVG Exo injection, confirming that both approaches induced the central effects of FGF1 (Figure 7E,F).

To further evaluate the long‐term hypoglycemic efficacy of the formulation, we established a diabetic stroke model. Treatment was initiated immediately after PT model establishment, with free FGF1 and FGF1‑loaded exosomes administered at a dose of 0.5 mg kg^−1^ on days 0, 7, 14, and 21. Body weight, food intake per mouse, and random blood glucose levels were monitored weekly. On day 28 post‐treatment, intraperitoneal insulin tolerance tests (ipITT) and intraperitoneal glucose tolerance tests (ipGTT) were conducted. Mice were then euthanized, and pancreatic islets were isolated for immunofluorescent staining of insulin. During the treatment period, no significant weight loss was observed in any group following administration, and food intake remained unaffected, indicating satisfactory safety. We also verified that FGF1‐RVG Exo did not lower blood glucose levels in normoglycemic mice (Figure S15A–C). As shown in Figure 7G, FGF1‐RVG Exo significantly reduced blood glucose levels by day 28 post‐treatment. In contrast, mice in the free FGF1 and FGF1‐Exo groups exhibited fluctuations in blood glucose levels despite weekly dosing, failing to achieve sustained and stable glycemic control. Additionally, FGF1‐RVG Exo improved glucose tolerance and insulin sensitivity in diabetic stroke mice (Figure 7H–K).

Extensive studies have demonstrated that repeated peripheral administration of FGF1 increases the density of insulin‑secreting β‑cells and enhances insulin secretion within pancreatic islets, whereas centrally administered FGF1 only transiently increases β‑cell density without promoting insulin secretion [35]. On day 28, pancreatic tissues from each group were isolated and subjected to insulin fluorescence staining. The results revealed that all FGF1‐loaded formulations (FGF1, FGF1‐Exo, and FGF1‐RVG Exo) increased insulin secretion in damaged islets, effectively elevated β‐cell density, and reduced serum insulin levels. In contrast, the blank exosome and PBS groups showed no such effects (Figure S16). These data indicate that FGF1‐RVG Exo not only crosses the blood‐brain barrier to exert sustained central hypoglycemic effects but also confers peripheral benefits by increasing pancreatic β‐cell density and promoting insulin secretion. This dual advantage of possessing both central and peripheral FGF1 effects enhances the clinical applicability potential of FGF1‐RVG Exo, although further validation through subsequent experiments is warranted.

### 2.6 FGF1‐RVG Exo Alleviates Brain Tissue Damage and Improves Post‐Stroke Motor Functional Recovery

Next, we examined the effect of FGF1‐RVG Exo on the functional recovery of PT mice. Pre‐trained T2DM mice were subjected to PT followed by intravenous administration of different formulations at 1 h after surgery. To assess motor function recovery, behavioral assessments were conducted at designated time points according to the schedule (cylinder test, rotarod test and grid walking test): pre‐stroke to establish a baseline, and then at regular intervals following PT surgery (days 3, 7, 14, 21 and 28) to monitor motor function recovery (Figure 8A). The mNSS score is widely used to evaluate the overall neurological recovery of rodents following stroke. After stroke induction, an increase in the mNSS score was observed in all groups, indicating worsening neurological dysfunction. Four weeks later, the mNSS score was significantly lower in the FGF1‐RVG Exo group than in the PBS group (Figure S17A). This indicated a favorable prognosis for stroke. In the cylinder test, intravenous injection of FGF1‐RVG Exo significantly reduced bias compared with PBS injection on days 3 and 7 after PT modelling induction (Figure 8B). All mice underwent rotarod training for three days prior to PT modelling and successfully completed the adaptation training (Figure S17B). Following stroke induction, a decline in motor performance was evident in both the treatment and PBS groups in the rotarod test (−68% in the PBS group, −73% in the Blank Exo group, −65.5% in the FGF1 group, −57.3% in the FGF1‐Exo group and −44.5% in the FGF1‐RVG Exo group). Four weeks after stroke induction, mice that received FGF1‐RVG Exo showed improved recovery in the rotarod test compared to the PBS group (Figure 8C). Furthermore, the intravenous injection of FGF1‐RVG Exo significantly decreased the rate of foot faults compared to the PBS injection on days 7, 14, 21, and 28 after the PT model was induced in the grid walking test (Figure 8D).

**FIGURE 8:**
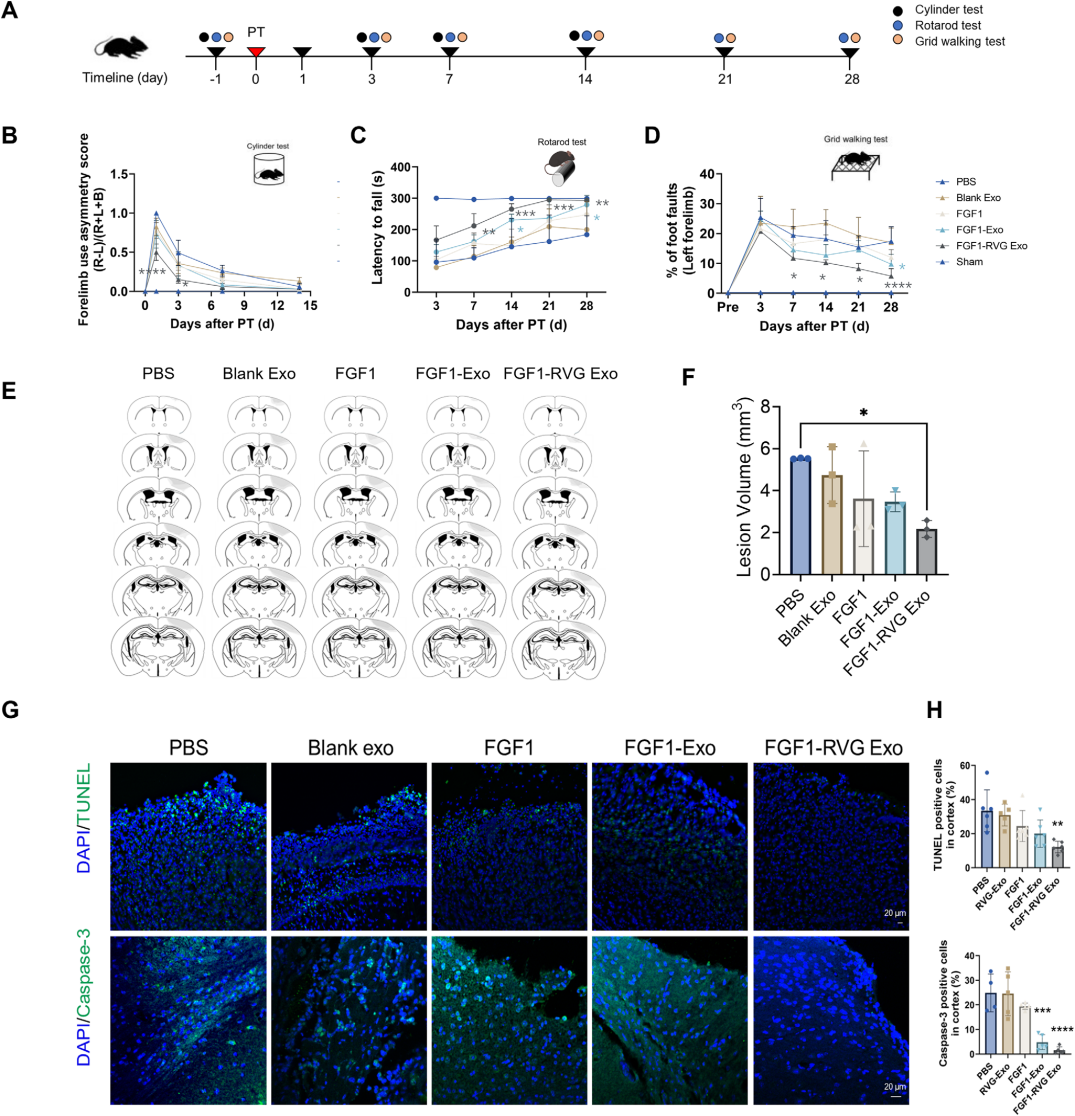
FGF1‐RVG Exo Treatment Effectively Improved Impaired Motor Function and Reduce Damage to Neurons. (A) Schematic of FGF1‐RVG Exo administration and behavioral analysis. (B) Cylinder test on 3, 7, 14 days after treatment.. (C) Rotarod test result on 3, 7, 14, 21, 28 days after treatment. (D) Grid walking test result on 3, 7, 14, 21, 28 days after treatment. Data are represented as mean ± SD (n = 5 per group). (E,F) Lesion reconstruction. Darker shades represent more overlap between animals. Data are represented as mean ± SD (n = 3 per group, ^*^ *p* <0.05). (G,H) Representative images with TUNEL staining and Caspase‐3 staining showing the number of apoptotic cells in the peri‐infarct cortex at day 28 after PT induction. Scale bars, 50 µm. Data are represented as mean ± SD (n = 3). (B), (C), (D) are analyzed by two‐way RM ANOVA; (F) and (H) are analyzed by one‐way ANOVA; ^**^ *p* <0.01; ^***^ *p* <0.001; ^****^ *p* <0.0001 vs. corresponding PBS group.

Following a stroke, secondary damage caused by factors such as the inflammatory storm can lead to the continuous spread of the infarct zone to surrounding areas, resulting in an increase in infarct volume. To this end, we performed Nissl staining on brain sections and compared the stroke lesion volumes in each group following treatment. Lesion size significantly decreased in the FGF1‐RVG Exo group compared with the PBS group, but did not differ between other groups (Figure 8E,F). To assess apoptosis in the injured area following treatment, we performed apoptosis and caspase 3 staining on brain tissue sections 28 days post‐treatment and calculated the corresponding number of positive cells. The results indicated that both the FGF1‐Exo group and the FGF1‐RVG Exo group significantly reduced the number of apoptotic cells at the injury site, but the FGF1‐RVG Exo group achieved the most effective treatment efficacy (Figure 8G,H).

### 2.7 FGF1‐RVG Exo Promotes Post‐Stroke Vascular Repair

Previous studies have proved that neurogenesis and angiogenesis are coupled processes after ischemic stroke, which are critical in improving post‐stroke neurological functional recovery [36]. Research indicates that FGF1 can promote angiogenesis in chicken choroid (CAM) via the AKT/PKB signaling pathway [37]. To determine whether FGF1‐RVG Exo can promote vascular regeneration following stroke in a type 2 diabetic model, we monitored blood flow changes in each group on days 1,7 and 14 using a doppler speckle flowmeter (Figure 9A) and calculated blood perfusion in the injury area on each time point (Figure 9B). We found no statistically significant differences in blood perfusion among the groups. However, on day 14, we observed that blood perfusion in the injury area of the FGF1‐Exo group and FGF1‐RVG Exo was significantly higher than PBS groups. Notably, there was no statistically significant difference in blood perfusion between the FGF1 group and the PBS group (Figure 9B), indicating that exosomes loaded with FGF1 are effective in promoting the recovery of cerebral blood flow in mice.

**FIGURE 9:**
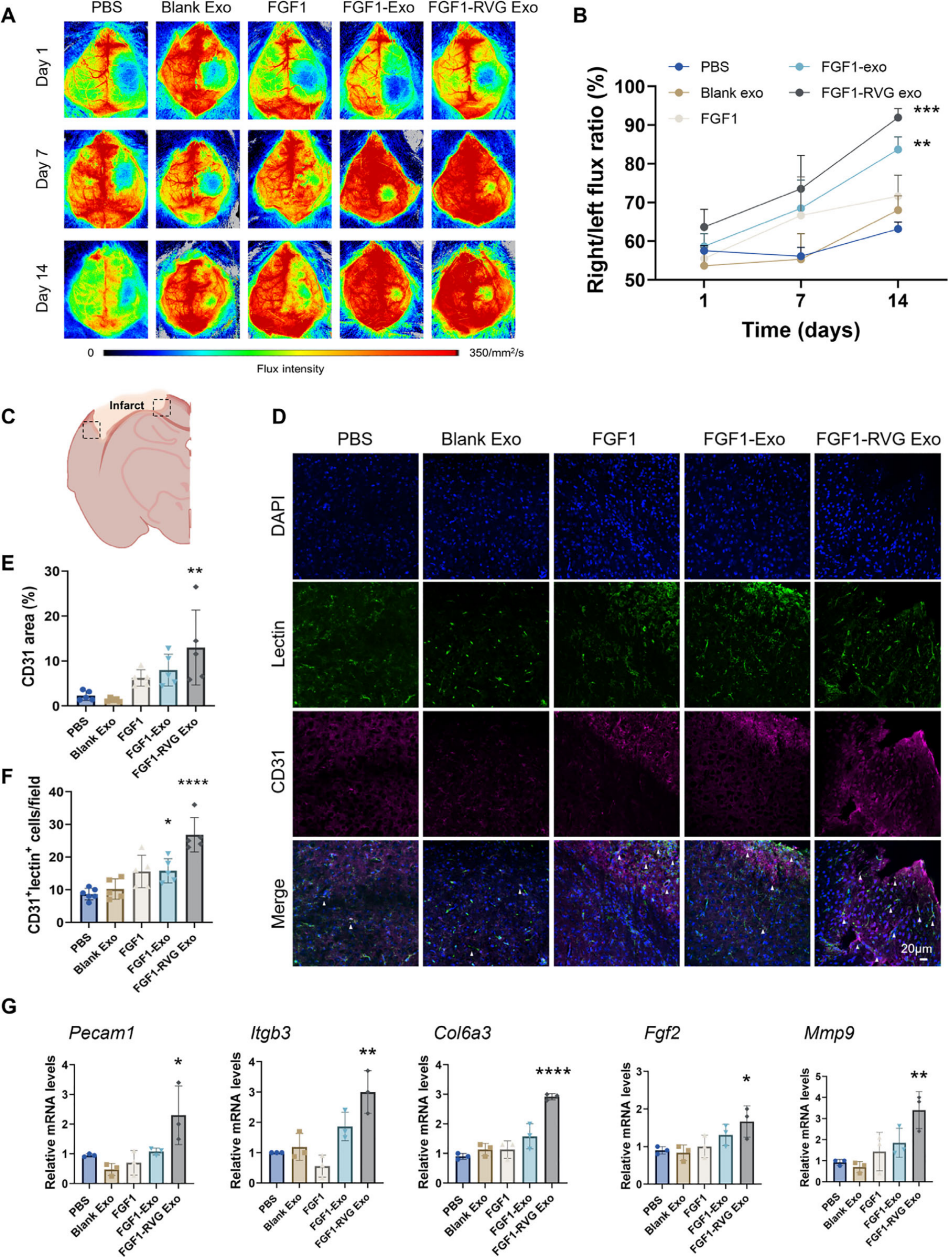
FGF1‐RVG Exo promoted vascular repair during stroke recovery. (A) Schematic representation of cerebral blood flow measurements in the same animal for each group at sequential time points for animals after stroke. (B) Statistics of peri‐infarct blood flow on day 1, 7 and 14. Data are shown as mean ± SD, n = 3, Statistical analyses were performed by two‐way repeated ANOVA test, with Greenhouse‐Geisser correction applied to adjust the degrees of freedom due to violation of sphericity (as indicated by Mauchly's test of sphericity. ^**^p <0.01; ^***^p <0.001 compared with PBS group. (C) Schematic diagram of the imaging position of blood vessels. (D‐F) Representative images with CD31 and lectin staining showing blood vessels in the peri‐infarct cortex at day 14 after PT in mice, followed by the analysis of CD31 positive area, double‐positive cell numbers per area (n = 5, Data are shown as mean ± SD. Statistical analyses were performed by one‐way ANOVA with Bonferroni's multiple‐comparison test. ^*^p <0.05; ^**^p <0.01; ^****^ *p* <0.0001 compared with PBS group). (G) qPCR analysis of angiogenesis‐related gene expression (n = 3, Data are shown as mean ± SD. Statistical analyses were performed by one‐way ANOVA with Bonferroni's multiple‐comparison test. ^*^p <0.05; ^**^p <0.01; ^****^p <0.0001 compared with the PBS group).

To assess tissue neovascularization, photothrombotic infarction was induced in T2DM mice. To label proliferating endothelial cells, 5‐ethynyl‐2'‐deoxyuridine (EdU) was administered daily from day 7 to day 9 post‐stroke (Figure S18A). At 14 days post‐stroke, brain sections were co‐stained for EdU and CD31. Quantitative analysis revealed that the number of CD31^+^EdU^+^ cells was significantly higher in the FGF1‐RVG Exo group compared to the PBS control group (Figure S18B,C). To evaluate functional blood perfusion, we performed Tomato lectin perfusion, which binds specifically to glycoproteins on the luminal surface of vascular endothelial cells, allowing for the visualization of functional blood vessels. Coronal brain sections were subjected to CD31/lectin immunofluorescence staining at day 14 post‐stroke. Our results showed that the FGF1‐RVG Exo group exhibited a significant increase in the CD31^+^ area. Furthermore, the number of CD31^+^lectin^+^ co‐localized cells (indicating functional proliferated vessels) was significantly increased in both the FGF1‐Exo and FGF1‐RVG Exo groups (Figure 9C–F).

Research indicates that angiogenesis involves a sprouting process, in which integrin subunit beta 3 (*Itgb3*), endothelial cell‐specific molecule 1 (*Esm1*), fibroblast growth factor 2 (*Fgf2*), and matrix metalloproteinase 9 (*Mmp9*) play key roles. In contrast, platelet/endothelial cell adhesion molecule 1 (*Pecam1*), collagen type IV alpha 2 chain (*Col4a2*), and collagen type VI alpha 3 chain (*Col6a3*) are primarily involved in vascular maturation. Additionally, Rho‐associated coiled‐coil‐containing protein kinase 1 (*Rock1*) and Ras homolog family member A (*Rhoa*) participate in blood‐brain barrier repair and the regulation of hypoxic responses after stroke [38]. To further investigate the effect of FGF1‐RVG Exo on these vascular repair programs, we euthanized the mice on day 14 and isolated peri‐infarct brain tissue for RNA extraction and qPCR analysis. Our results showed that in the FGF1‐RVG Exo group, the mRNA expression levels of sprouting‐related genes (*Itgb3*, *Fgf2*, and *Mmp9*) and maturation‐related genes (*Pecam1* and *Col6a3*) were significantly higher than those in the PBS group (Figure 9G). In contrast, no significant differences were observed among the groups in the expression levels of *Rock1*, *Esm1*, *Col4a2*, or *Rhoa* (Figure S18D). Taken together, these findings suggest that FGF1‐RVG Exo facilitates functional vascular recovery after injury by simultaneously promoting angiogenic sprouting and vascular maturation, thereby enhancing neovascularization in the peri‐infarct region.

### 2.8 FGF1‐RVG Enhances Long‐Term Functional Connectivity in Cognitive‐Associated Regions Post‐Stroke

T2DM has been associated with morphological alterations in hippocampal neurons and impaired learning function [39]. Nissl staining revealed that in the PBS group, neurons in the hippocampal CA1 region exhibited widespread loss, nuclear degeneration, indistinct cell membranes, and sparse arrangement. In contrast, diabetic stroke mice treated with FGF1‐RVG Exo showed normal neuronal morphology, characterized by large round nuclei and dense arrangement, with outcomes superior to those in the FGF1 group (Figure 10A). The cortex is a non‐neurogenic region, and its neurogenic potential becomes minimal in adulthood [40]. Previous studies have demonstrated the extremely limited generation and survival of adult‐born neurons after stroke [41]. To investigate the long‐term effects of FGF1‐RVG Exo on neuronal survival post‐stroke, we performed immunofluorescence staining for NeuN (a marker of mature neurons) on coronal brain sections at day 28 post‐stroke. NeuN^+^ cells were significantly increased in the stroke cavity of the FGF1‐loaded groups (Figure 10B,C). Ischemic stroke is known to induce substantial myelin loss in the ischemic region, accompanied by neuronal loss and axonal degeneration [42]. To evaluate the impact of treatment on myelin integrity, we examined myelin changes within the injured brain region. In the PBS group, extensive loss of MBP‐positive myelin and a reduction in the number of NF200‐positive axons were observed within the lesion area and penumbra. In contrast, treatment with FGF1‐loaded formulations resulted in a marked increase in both MBP‐positive myelin area and NF200‐positive axon count, with the expansion of the myelin area being particularly prominent. These findings suggest that the treatment effectively promotes remyelination and contributes to the recovery of neurological function (Figure 10D–F).

**FIGURE 10:**
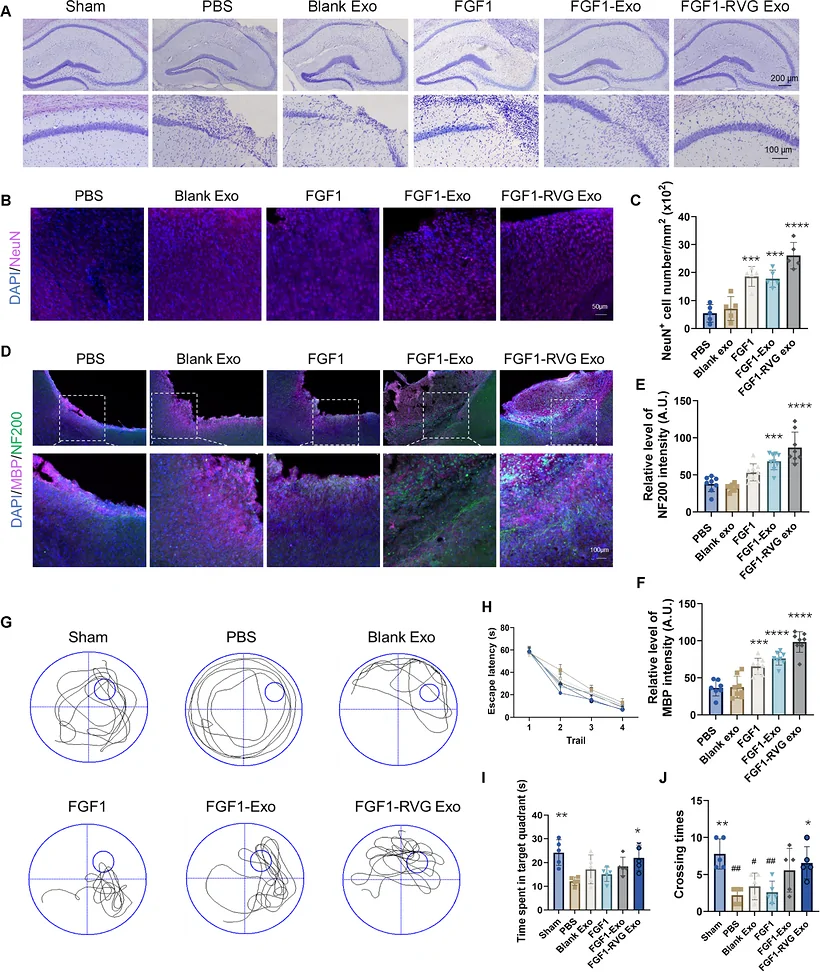
FGF1‐RVG Exo promoted the recovery of cognitive functions. (A) Schematic representation of Nissl staining for each group after treatment. (B,C) Representative images of NeuN staining for each group after treatment. (D–F) Representative images of MBP, NF200 staining for each group after treatment. (n = 3, Data are shown as mean ± SD. Statistical analyses were performed by one‐way ANOVA with Bonferroni's multiple‐comparison test. ^**^p <0.01; ^***^p <0.001; ^****^p <0.0001). (G) Representative images of the Morris water maze test represented after treatment. (H) Escape latency; (I) Time spent in the targeted quadrant; (J) Number of platform crossing. (Data are shown as mean ± SD, n = 5. ^*^p <0.05,^**^ *p* <0.01 compared with PBS group, *^#^p* <0.05, ^##^ *p* <0.01 compared with Sham group).

Given the critical role of the hippocampus in memory and learning, we subsequently performed a Morris water maze test to evaluate the learning and memory abilities of mice in each group. After four days of training, the platform was removed on the fifth day. We recorded the latency times during the training days, as well as the number of platform crossings and the time spent in the target quadrant on the fifth day for each group (Figure 10G–J). The number of platform crossings in the PBS and Blank Exo groups was significantly lower than that in the Sham group. Following administration of FGF1‐RVG Exo, both the number of platform crossings and the time spent in the target quadrant were significantly higher than those in the PBS group, whereas mice treated with FGF1 alone showed no statistically significant difference compared to the PBS group. These results indicate that FGF1‐RVG Exo promotes the recovery of learning and memory abilities in stroke‐affected mice, with an effect superior to that of FGF1 alone. Collectively, these findings demonstrate that administration of FGF1‐RVG Exo prevents cortical neuron loss, enhances myelination and axonal integrity in the injured region, and promotes neuronal maturation, long‐term survival, and cognitive recovery after diabetic stroke.

Finally, we evaluated the biosafety of FGF1‐RVG Exo at the administered dose. As shown in Figure S19, H&E‐stained sections of major organs, including the heart, liver, spleen, lung, and kidney, showed no obvious pathological abnormalities following FGF1‐RVG Exo treatment. These findings indicate that FGF1‐RVG Exo exhibited a favorable preliminary biosafety profile under the current treatment regimen.

## 3 Discussion

Diabetic stroke represents one of the most serious manifestations of cerebrovascular disease, with approximately 33% of stroke patients affected by diabetes and experience a poor prognosis [43]. Moreover, diabetic patients with ischemic stroke who undergo mechanical thrombectomy often have poor outcomes despite successful recanalization [44], highlighting the urgent need for adjunctive pharmacological interventions targeting microcirculation and metabolism. Accumulating evidence suggests that FGF1 confers dual therapeutic benefits for both diabetes and ischemic stroke, positioning it as a highly promising candidate for the treatment of diabetic stroke [19, 21, 45]. In this study, we first confirmed that FGF1 expression is closely associated with the pathogenesis of diabetic stroke and developed a brain‐targeted delivery system, FGF1‐RVG Exo, to overcome the challenges associated with delivering FGF1 to the brain (Figures 2, 3, 4, 5). Our results demonstrated that intravenously injected FGF1‐RVG Exo effectively crosses the BBB and delivers FGF1 directly to the injured brain region, achieving enhanced permeability and retention without the need for peripheral multi‐frequency administration or central invasive injection. Remarkably, a single intravenous dose of FGF1‐RVG Exo produced a sustained hypoglycemic effect comparable to that of intracerebroventricular FGF1 administration. Furthermore, weekly administration of FGF1‐RVG Exo effectively preserved neuronal survival in the injured area, reduced infarct volume, promoted neovascularization and functional blood vessel formation, and improved both motor function and cognitive recovery (Figures 8, 9, 10). Collectively, these findings demonstrate that FGF1‐RVG Exo represents a promising strategy for simultaneously addressing the core pathological features of diabetic stroke‐namely, glycemic dysregulation and post‐stroke neurological damage, thereby achieving dual therapeutic benefits.

Accumulating evidence suggests that FGF1 exerts therapeutic effects in metabolic diseases, including obesity, type 2 diabetes, and related complications. To investigate the association between FGF expression and diabetic stroke, we previously conducted a bioinformatics analysis of the GSE26168 dataset, which comprises blood samples from patients with diabetes or impaired fasting glucose. The results revealed that, among the 23 FGF family genes, only FGF1 was associated with diabetes or impaired glucose tolerance, and its expression levels were significantly downregulated in the blood of diabetic patients. Furthermore, analysis of RNA transcriptomic sequencing data from brain endothelial cells of post‐stroke mice showed that FGF1 expression was significantly downregulated during the acute phase of stroke, with gradual recovery during the subacute and recovery phases. Meanwhile, we also verified the downregulated protein and mRNA expression of FGF1 in the animal model. These findings are consistent with those of previous studies [46, 47]. Collectively, these findings demonstrate a close association between FGF1 and diabetic stroke. Notably, endogenous FGF1 levels exhibit a declining trend with disease progression, suggesting that timely exogenous supplementation during the acute phase of stroke may exert critical therapeutic effects. Previous studies have shown that frequent peripheral injections or intranasal administration of FGF1 during the acute phase effectively modulates post‐stroke microglial polarization, promotes neurogenesis, regulates glucose and lipid metabolism, and consequently reduces infarct volume while enhancing angiogenesis. However, these studies invariably required continuous daily or every‐other‐day dosing (0.5 mg kg^−1^) [19, 21, 39]. Such high‐frequency administration regimens may pose safety concerns owing to the mitogenic activity of FGF1 and impose a significant burden on patients, thereby hindering clinical translation. In contrast, the FGF1‐RVG Exo formulation developed in this study achieves efficient brain‐targeted delivery of FGF1. A single intravenous dose produces a sustained central hypoglycemic effect comparable to that of intracerebroventricular injection and effectively increases pERK levels in the ARC region. These findings collectively indicate that this formulation holds promise for overcoming the challenges associated with brain delivery of FGF1, reducing dosing frequency, and thereby enhancing treatment safety.

RVG peptides exhibit high neurotropism and can deliver molecules to neurons via receptor‐mediated transcytosis. RVG‐modified exosomes not only confer central nervous system (CNS) targeting capabilities but also, owing to their lipid membrane structure, efficiently mediate the intercellular transport of bioactive molecules, making them ideal carriers for brain‐targeted drug delivery [48, 49]. Traditional engineered exosome delivery strategies have primarily focused on encapsulating therapeutic proteins within the vesicular lumen‐a process largely dependent on the intrinsic sorting mechanisms of exosomes‐and delivering the cargo to the cytoplasm or perinuclear region of target cells via the intracellular transport pathway of “membrane recognition–endosomal uptake–lysosomal escape.” Most previous studies have demonstrated the therapeutic efficacy of engineered exosomes in diseases such as ischemic stroke and Alzheimer's disease without investigating the precise amount of active proteins encapsulated within the vesicles [50, 51]. Given that the endogenous cargo of exosomes is inherently complex, comprising various RNAs (including mRNA, miRNA, and lncRNA) and proteins, it is challenging to determine which components are primarily responsible for the therapeutic effects. The present study, therefore, focuses on the delivery of active proteins with strong clinical translational potential to the brain, representing a distinct contribution of this work.

We initially attempted to generate FGF1‐loaded RVG exosomes via co‐transfection with two plasmids; however, the resulting exosomes contained only very low levels of active FGF1. Moreover, the efficiency of dual‐plasmid transfection is generally lower than that of single‐plasmid transfection. Accordingly, we subsequently explored an exogenous loading strategy. This approach not only preserves the intrinsic biological activity of FGF1 and the natural properties of exosomes but also exhibits excellent capacity to cross biological barriers, offering clinical advantages that are not achievable with most synthetic nanomaterials or existing clinical interventions. Furthermore, as a typical membrane‐bound growth factor, the biological function of FGF1 primarily relies on its binding to FGFR receptors on the surface of target cells to activate intracellular signaling pathways, thereby exerting its pharmacological effects without the need for cellular internalization. Consequently, the prevailing “cytoplasmic delivery”‐oriented strategy is fundamentally incompatible with the “membrane‐surface action” mechanism of FGF1. To date, few studies have reported exosome‐mediated surface‐anchored delivery strategies for membrane‐bound proteins, particularly in the context of CNS disease treatment, highlighting a significant research gap. In this study, we innovatively employed engineered exosomes as transport vehicles and explored three different methods to load FGF1 onto the vesicle surface. Although the loading efficiency was modest, the FGF1‐RVG Exo prepared through exogenous incubation successfully delivered FGF1 across the blood–brain barrier to the damaged brain region, thereby exerting sustained pharmacological effects, including hypoglycemic activity, promotion of vascular regeneration, and facilitation of neural functional recovery.

Drug delivery to the brain remains a significant challenge. Our findings support an exosome‐mediated paracrine growth factor delivery model, in which molecules are transferred from nanocarriers to the brain without requiring significant nanoparticle internalization. We propose that engineered RVG exosomes enhance affinity for the BBB surface, thereby improving brain‐targeted delivery of FGF1. This is supported by the observation that RVG Exo increases FGF1 enrichment in the stroke‐injured region, likely through specific binding of RVG peptides to acetylcholine receptors, which is consistent with previous reports [52, 53]. These results suggest that engineered RVG exosomes hold great promise as brain‐targeted drug delivery vectors.

Nevertheless, this study has notable limitations. Loading therapeutic proteins as cargo is more challenging than loading small molecules or RNA due to issues involving sorting efficiency, expression stability, and maintenance of protein conformation and activity. Soluble proteins are typically hydrophilic and possess large molecular weights, making it difficult for them to cross the exosome lipid bilayer. Multiple studies have clearly shown that simple incubation allows proteins to adsorb onto the surface of exosome membranes, but the loading efficiency is generally low. Although electroporation has been used to load proteins such as p53 and antibody fragments in experimental settings, this approach is fundamentally limited by damage to the exosome membrane and protein denaturation. Ultrasound and freeze‑thaw methods can alter exosome membrane particle size and membrane protein structure, while the addition of permeabilizing agents such as saponin may cause residual permeation and structural changes to the membrane [54, 55, 56]. In our study, we opted for three relatively mild exogenous loading methods instead of electroporation, as only mild preparation strategies were considered feasible to preserve the biological activity of FGF1. In principle, FGF1 can only bind non‑covalently to exosomal membrane proteins, resulting in low loading efficiency and potential off‑target risks if the binding is insufficiently stable. Nonetheless, our study preliminarily confirms the feasibility of this approach for delivering therapeutic proteins, although both delivery efficiency and encapsulation strategy warrant further improvement. Therefore, we believe that post‑loading methods are not the optimal pathway for engineered exosome‑based protein delivery systems, but are only suitable for proteins with well‑established purification processes and whose targets are located on the cell surface or outside the cell. Once the therapeutic effect is confirmed, introducing the coding sequence of the therapeutic protein into donor cells, fusing the target protein with an exosome scaffold protein, and designing the protein to be actively sorted and expressed at the correct location based on its target may represent a carrier construction strategy with greater clinical translational value. For example, PTGFRN has been reported to serve as an EV scaffold for high‑density display of various proteins on its surface [57]. Therefore, in future studies, we aim to further engineer both FGF1 and the exosome membrane to achieve improved drug loading and stable enrichment.

In conclusion, our study first confirmed a strong correlation between FGF1 and disease progression in diabetic patients with ischemic stroke, suggesting that timely administration of FGF1 during the acute phase may hold significant therapeutic promise. We further observed that mice with diabetic stroke exhibited more severe neurological deficits than their normoglycemic counterparts, supporting the urgent need for effective interventions. To address this, we developed an engineered exosome‐based platform by generating RVG‐overexpressing exosomes and subsequently loading FGF1 via co‐incubation to produce FGF1‐RVG Exo. This construct ensures the attachment of FGF1 to the membrane surface of RVG exosomes, thereby improving accumulation in the infarct area and significantly enhancing drug penetration in vivo. Consequently, FGF1‑RVG Exo exerts multifaceted pharmacological effects in diabetic stroke mouse models, including sustained glycemic regulation, restoration of impaired pancreatic function, promotion of vascular regeneration, and facilitation of motor and cognitive recovery. Notably, these therapeutic benefits were achieved with a once‑weekly administration frequency while maintaining a favorable safety profile. Collectively, this modified exosome‑based engineering approach provides a streamlined and versatile platform for the brain‑targeted delivery of therapeutic proteins such as FGF1.

## 4 Experimental Section

### 4.1 Animals

All animal experiments were conducted at the Laboratory Animal Services Center (LASC) of Wenzhou Medical University in compliance with local regulations for animal research. Male specific‑pathogen‑free BALB/c mice (aged 8 weeks) were purchased from GemPharmatech Co., Ltd (Jiangsu, China; laboratory animal license number: SCXK (Su) 2023‑0009). Mice were housed under a 12‑h light/dark cycle with free access to food and water. The animal study protocol was reviewed and approved by the Laboratory Animal Ethics Committee of Wenzhou Medical University (Ethics approval number: xmsq2023‐0463). All procedures were performed in accordance with the Guidelines for the Care and Use of Laboratory Animals of Wenzhou Medical University, and all efforts were made to minimize animal suffering.

### 4.2 Cell Culture

All cells were cultured in DMEM supplemented with 10% fetal bovine serum (FBS) and 1% penicillin‐streptomycin in a humidified atmosphere at 37°C with 5% CO_2_.

### 4.3 Bioinformatics Analysis

Gene Expression Omnibus (GEO) Series GSE26168 dataset was downloaded from the GEO database of the National Center for Biotechnology Information.

### 4.4 Blood Flow Imaging

Blood flow was imaged through a cranial window with laser doppler flowmetry, a label‐free, optical method. Mice were anesthetized with isoflurane. Body temperature was maintained with a heated pad during the experiment. Mice were head‐fixed under the guidance of the red light and exposed the skull. Backscattered light was collected by a CCD camera. Images were exported to Image J to measure the area of the injury region. Blood flow was measured both the ipsilateral and contralateral regions over time.

### 4.5 Induction of Diabetes and Blood Glucose Monitoring

Diabetes was induced 8 weeks after chronic high‐fat diet (HFD) feeding. A 5‐d induction protocol was followed over consecutive days as described before [21]. On each day, mice were deprived of food for 12 h, followed by intraperitoneal injection of STZ (40 mg kg^−1^) dissolved in 50 mm (pH = 4.5) sodium citrate buffer. 5 days after injection, fasting blood glucose levels of each mouse were measured to observe whether the type 2 diabetic model was successfully induced. For simplicity, mice that exhibit STZ‐induced type 2 diabetes will be referred to as “diabetic” in the remainder of the paper.

### 4.6 Photothrombotic Stroke Induction

A photothrombotic stroke was induced in the left sensorimotor cortex using Rose Bengal (30 mg kg^−1^, Sigma) and cold light (KL 1600 LCD, Schott) as previously described [58, 59]. Briefly, mice were anesthetized with isoflurane in medical air (2% induction, 1.5% maintenance) and were placed on a heating pad to maintained body temperature during the experiment. The head was secured in a stereotaxic frame, the fur on top of the head was shaved, and a midline incision was made along the scalp. Rose Bengal was intraperitoneally injected 5 min before irradiation. A black mold was mounted on the fiber optic bundle in order to produce an irradiation area of a circle with a diameter of 2 mm. The fiber bundle was then positioned on the skull 1 mm lateral from the bregma, and the green light was turn on at 75% for 15 min.

### 4.7 FGF1‐RVG Exo Preparation and Characterization

The FGF1‐RVG Exo was fabricated with the following three steps: (i) Transfection of RVG‐lamp2b plasmid into HEK293T cells; (ii) Collection of RVG exosomes from 293T cells; (iii) Exogenous loading of FGF1 onto exosomes. For FGF1, 1 mg of FGF1 was fully dissolved in 0.5 mL of sterile PBS. Before FGF1 loading, each exosome batch was individually characterized in terms of particle concentration and total exosomal protein content using NTA and the Bradford assay, respectively. This characterization allowed for a precise quantification, estimating approximately 2.20 ± 1.54 × 10^7^ exosomes per µg of exosome protein to be used in each experimental setup. This was particularly relevant since the same number of exosomes not always correspond to the same protein content. Therefore, we normalized for both the number and protein content. Subsequently, the exosomes were then divided into three groups and FGF1 loaded according to the following methods: (i) co‐incubation at 4°C overnight or 37°C for 12 h; (ii) ultrasound for 30 s under 20% frequency under five cycles; (iii) freeze at −80°C and thaw at 37°C for three cycles. Free FGF1 was removed by centrifugation through a 10‐KDa ultrafiltration at 5000 g for 20 min. The final mass ratio of FGF1: exosome was 1:2. Exosomes were observed under TEM.

### 4.8 Detection of Exosome Crossing the BBB Efficiency

We labeled exosomes with DiD dye, which is a red fluorescent protein (640 nm excitation/ 670 nm emission). 24 h post‐injection, the mice were euthanized, and the brain tissues were harvested and sectioned for examination. Confocal imaging was used to investigate the distribution of exosomes in brain tissues. In the mice injected with DiD‐labeled exosomes, significantly fluorescent intensity was observed in cortical injury areas.

### 4.9 Immunohistochemistry

For immunohistochemistry, animals were transcardially perfused with 0.9% NaCl. Brain tissues were then extracted, post‐fixed in 4% paraformaldehyde (PFA) for 6 h, and subsequently dehydrated in a sucrose gradient before being embedded in optimal cutting temperature (OCT) compound. Free‐floating brain sections were blocked in blocking solution (3% bovine serum albumin (BSA) with 0.3% Triton X‐100 in PBS) for 1 h at room temperature (RT), followed by incubation with primary antibodies overnight at 4°C. On the following day, sections were incubated with secondary antibodies for 1 h at RT, and nuclei were counterstained with DAPI. Images were acquired using a confocal laser‐scanning microscope (Olympus SpinSR, Japan).

The following primary antibodies were used: monoclonal mouse anti‐CD31 (1:200; Abcam), monoclonal rabbit anti‐NeuN (1:500; CST), polyclonal rabbit anti‐Caspase‐3 (1:200; Abcam), monoclonal mouse anti‐MBP (1:200; Santa Cruz), and monoclonal rabbit anti‐NF200 (1:200; Abcam). All secondary antibodies were purchased from Abcam. Alexa Fluor 488‐conjugated donkey anti‐rabbit, Alexa Fluor 647‐conjugated donkey anti‐rabbit, and Alexa Fluor 488‐conjugated donkey anti‐mouse antibodies were used to visualize primary antibody binding.

To evaluate cell proliferation at 2 weeks after treatment, EdU labeling was performed. EdU was dissolved in sterile PBS at a concentration of 5 mg mL^−1^ and administered via intraperitoneal injection at a dose of 50 mg kg^−1^ body weight at 7 days post‐stroke. Brain sections were incubated with a reaction mix buffer for 30 min. Sections were then washed with 0.1 m PBS and mounted with anti‐fluorescence quenching mounting medium containing DAPI.

### 4.10 Infarct Volumes

Sections ranging from 0.94 to −3.04 mm from bregma were performed Nissl staining at 0.5 mm intervals. The non‐lesioned area of the infarcted hemisphere and the non‐lesioned contralesional hemisphere were outlined using ImageJ, and infarct volumes were calculated by integration as previously described [60].

### 4.11 Statistical Analysis

All data were analyzed using GraphPad Prism (version 8.0). Normality was assessed using the Shapiro–Wilk test. For comparisons between two normally distributed groups, an unpaired two‑tailed t‑test was used. For comparisons involving three or more groups, one‑way analysis of variance (ANOVA) followed by Tukey's post hoc test was applied. For behavioral tests, blood glucose monitoring, Doppler flowmetry, and the Morris water maze test, two‑way repeated measures ANOVA was used with Greenhouse–Geisser correction when sphericity was violated, followed by Sidak's post hoc test. Data are shown as mean ± SD, except for histograms of non‑normal data (medians with individual points). Statistical significance: *p* <0.05.

### 4.12 AI Assistance Disclosure

Grammar and typos were corrected with the assistance of ChatGPT (OpenAI). No content generation or data analysis was conducted using AI.

## Author Contributions

L.L., B.S., and X.L. designed the experiments. B.S., C.Z., J.W., X.W., X.Z., and S.C. performed the experiments. L.L., B.S., and J.W. wrote the manuscript. C.Z. and X.L. edited the manuscript. All authors provided critical feedback and approved the final version of the manuscript.

## Funding

This work was supported by the National Natural Science Foundation of China (No. 82372149), the “Pioneer” and “Leading Goose” R&D Program of Zhejiang Province (No. 2025C02152).

## Conflicts of Interest

The authors declare no conflicts of interest.

## Supporting information




**Supporting File**: advs77064‐sup‐0001‐SuppMat.docx.

## Data Availability

The data that support the findings of this study are available from the corresponding author upon reasonable request.
