# Supplementary material for: Engineered Brain‐Targeted Exosomes Delivering FGF1 for Sustained Glycemic Regulation and Multitarget Neurovascular Protection in Diabetic Stroke
Source: Adv Sci (Weinh). 2026 Aug 11:e77064. Online ahead of print. doi: 10.1002/advs.77064 (PMC13460273; doi:10.1002/advs.77064)
Supplement: Supplementary file 1 [file ADVS-9999-e77064-s001.docx]

Supporting Information

**Engineered Brain-Targeted Exosomes Delivering FGF1 for Sustained Glycemic Regulation and Multitarget Neurovascular Protection in Diabetic Stroke**

Bixin Shen^1,2^, Chengxiang Zhang^1^, Junhui Wang^1^, Xichong Zhong^1^, Xue Wang^1^, Shihao Chen^1^, Xiaokun Li*^1,2^, Li Lin*^1,2^

Figure S1. *FGF1* RNA expression in human normal tissue plotted transcripts per million (TPM). Data were obtained from Human Protein Atlas Dataset available from proteinatlas.org.

Figure S2. The diabetic milieu hindered recovery of motor function following stroke in these mice.

(A) Grid walking test results for left forelimb and left hindlimb in mice. Data are shown as mean ± SD. n = 5, ^*^*p* < 0.05, ^***^*p* < 0.001 and ^****^*p* < 0.0001. ns, not significant. (B) mNSS scoring results. Data are shown as mean ± SD. n = 5, ^****^*p* < 0.0001. (C) Grip strength test results. Data are shown as mean ± SD, n = 6, *^**^p* < 0.01, *^****^p* < 0. 0001.Statistical significance was assessed by one-way ANOVA followed by Tukey’s multiple comparisons test.

Figure S3. Particle concentration (A) and size distribution (B) of exosomes with and without Triton X-100.

Figure S4. The A260/A280 ratio of exosomes. Data are presented as mean ± SD (n = 3). *^*^p* < 0.05. Statistical significance was assessed using a two-tailed Student’s t-test.

Figure S5. Determination and analysis of the penetrative performance of RVG Exo through blood brain barriers. (n=3, Data are shown as mean ± SD. Statistical analyses were performed by one-way ANOVA with Bonferroni’s multiple-comparison test. *^**^p* < 0.01; *^***^p* < 0.001; *^****^p* < 0.0001).

Figure S6. Preparation and characterization of FGF1-Exo from FGF1-overexpressed HEK293T cells. (A) HEK293T cells are transfected with the FGF1 plasmid and RVG plasmid. (B) Cells were visualized by mCherry and GFP fluorescence. (C) Statistics of fluorescence intensity after transfection. n=3, Data are shown as mean ± SD. (D) Flow cytometry statistics of double positive cell portions.

Figure S7. (A) Fluorescence colocalization of mother cells after transfection with both plasmids. Scale bar = 10 μm. (B) WB detection of FGF1 content in cells and exosomes. (C) Standard curve for FGF1 content by ELISA. (D) The colocalization of RVG and FGF1 with exosome markers.

Figure S8. The encapsulation rates of FGF1 in FGF1-RVG prepared by three methods.

Figure S9. Dynamic light scattering measurements for hydrodynamic diameter of FGF1-Native Exo and FGF1-RVG Exo. (A) Particle size (B) PDI and (C) Zeta potential.

Figure S10. (A) WB analysis of FGF1 in upper and lower chambers of filtration. (B) The colocalized fluorescence imaging of exosomes and FGF1. (C) Zero-order Kinetic Fitting of drug release curves.

Figure S11. (A) Ex vivo fluorescent images of various organs at 3 hours after the injection of DiD-labeled exosomes. (B) Semiquantification of the fluorescent signals in various organs excised from mice. (Data are shown as mean ± SEM. n=3, *^**^p* < 0.01, ns, not significant).

Figure S12. Representative confocal fluorescent microscopy images of the brain section with i.v. injections of free dye.

Figure S13. Colocalization analysis of cerebral vascular and FGF1-RVG Exo. Scale bar = 10μm

Figure S14. The quantification of the fluorescence intensity in cell uptake test. Statistical significance was assessed using a two-tailed Student’s t-test. Data are presented as mean ± SD (n = 3). *^*^p* < 0.05, *^**^p* < 0.01 was considered statistically significant. ns, not significant.

Figure S15. (A) Weekly Body weight monitoring after after i.v. injections of different formulations in diabetic stroke mice (n = 4). (B) Weekly food intake monitoring after i.v. injections of different formulations in diabetic stroke mice (n = 3). (C) Blood glucose levels monitoring in WT mice after administration of FGF1 and FGF1-RVG Exo (0.5 mg/kg i.v.). (n = 3). Data are expressed as mean ± SD. Statistical analyses were performed by two-way ANOVA test. *^**^p* < 0.01; *^***^p* < 0.001; n.s. not significant.

Figure S16. (A) H&E staining and immunofluorescence staining of insulin in pancreatic tissue after treatment. (B) average islet area (C) β-cell area (D) plasma insulin level was detected in each group after treatment. (n = 3-5 per group). Data are expressed as mean ± SD. Statistical analyses were performed by one-way ANOVA; *^*^p* < 0.05; *^**^p* < 0.01; *^***^p* < 0.001; ns, not significant.

Figure S17. (A) mNSS score of mice on day 3, 7, 14, 21 and 28 after PT injury (n = 5 per group). (B) Training result of rotarod test (n = 5 per group). Data are represented as mean ± SD. Data were analyzed by two-way ANOVA, *^*^p* < 0.05; *^**^p* < 0.01; *^***^p* < 0.001; *^****^p* < 0.0001 compared with PBS group.

Figure S18. (A) Experimental schematic diagram. (B) Representative images with CD31 and EdU staining. Scale bar = 50μm. (C) The quantification of CD31 and EdU double-positive cell per area. n = 3-4 per group. (D) qPCR analysis of angiogenesis-related gene expression. n=3, Data are shown as mean ± SD. Statistical analyses were performed by one-way ANOVA. *^**^p* < 0.01 compared with PBS group.

Figure S19. H&E-stained slice images of major organs (heart, liver, spleen, lung, and kidney) of treatment suggesting as FGF1-RVG Exo treatment was benign for these organs. Scale bar = 100μm.
